# Motivational power of future time perspective: Meta-analyses in education, work, and health

**DOI:** 10.1371/journal.pone.0190492

**Published:** 2018-01-24

**Authors:** Lucija Andre, Annelies E. M. van Vianen, Thea T. D. Peetsma, Frans J. Oort

**Affiliations:** 1 Research Institute of Child Development and Education, University of Amsterdam, Amsterdam, the Netherlands; 2 Work and Organizational Psychology, University of Amsterdam, Amsterdam, the Netherlands; University Hospital Jena, GERMANY

## Abstract

Future time perspective (FTP) may predict individual attitudes and behaviors. However, FTP research includes different FTP conceptualizations and outcomes which hinder generalizing its findings. To solve the inconsistencies in FTP research and generalize the magnitude of FTP as a driver of motivation and behavior, we conducted the first systematical synthesis of FTP relationships in three crucial life domains. Our meta-analyses of FTP studies in education (*k* = 28), work (*k* = 17), and health (*k* = 32) involved *N* = 31,558 participants, and used a conceptual model for grouping FTP constructs. To address different outcome types, we applied the Theory of Planned Behavior when coding the studies. FTP relationships with outcomes were small-to-medium, were generalizable across domains, and were strongest when the FTP construct included a mixture of cognition, behavioral intention, and affect and, in education, when the FTP measure was domain specific rather than general. There were cross-cultural differences in FTP-outcome relationships. The strength of the FTP-outcome types relationship varied for attitudes, perceived behavioral control, behavioral intention, and behaviors. The lowest effect sizes were found for FTP predicting actual behaviors in education, work, and health and between FTP and health attitudes. Theoretical implications of the findings and future research directions are discussed.

## Introduction

The future is not a result of a choice among alternative paths offered by the present, but a place that is created—created first in the mind and will, created next in activity. The future is not some place we are going to, but one we are creating. The paths are not to be found, but made, and the activity of making them changes both the maker and the destination [1].

The human capacity for contemplating the future is a basis of human motivation and behavior in everyday life. Imagine a student preparing for an exam who lacks insight into the ultimate purpose of his or her learning effort, an employee who lacks career perspective, or a person who is told to lose weight but who cannot envision what said weight loss could afford him or her. None of these people would be motivated to put much effort into their learning-, work-, or health-related endeavors, and perhaps the lack of a future time perspective (FTP) prevented them from planning and regulating their daily actions.

By definition, people’s motivational goals are situated in the future. Thinking about the future can concern the short term (e.g., visiting a dentist this afternoon) and the long term or more distant future (e.g., becoming a lawyer in 5 years [2]). Short-term goals are relatively easy to establish and are generally more concrete; they also tend to be strong drivers of motivation, self-regulation, and behavior [3–6]. Long-term goals, however, are often more difficult to determine and are more abstract because the future is further away; therefore, the psychological distance to the future is greater [7, 8]. The temporal distance of future, abstract goals (if they are set at all) could weaken goals’ potential capacity to motivate. At the same time, individuals who envision a distant future may be able to anticipate the future consequences of their present behavior [9–11]. In this paper, we pose a fundamental question regarding human motivation: *To what extent are people’s present motivation*, *intentions*, *and behaviors affected by their FTP?*

### Future time perspective

To achieve an in-depth understanding of human motivation and actions and reasons for those actions, the future merits a great deal more attention than it has received from the social sciences thus far [12]. From different theoretical perspectives, researchers have argued that thinking about the future influences human actions in the present.

As the concept of psychological time may include various aspects such are time succession, time duration, and time perspective [13], thinking about the future is a broad concept that has been approached from a variety of theoretical and empirical traditions and scientific fields [14]. One dominant research tradition is based on Lewin’s [15] and Frank’s [16] seminal theoretical framework of time perspective defined as ‘‘the totality of the individual’s views of his psychological future and psychological past existing at a given time” (p. 75). Based on this tradition, scholars have focused on individual differences in past, present, and future time perspective, and the extent to which individuals focus on one of these time perspectives when making decisions in different life domains. This research tradition has particularly focused on individuals’ propensity to reflect on the future and their general attitudes towards the future. Researchers investigated this FTP construct as a motivator for outcomes in different life domains such as education, work, health, and environment (e.g., [17–19]). For example, embedded in this research tradition, a meta-analysis by Milfont, Wilson and Diniz [18] has shown that FTP (as compared to past and present time perspective) is a motivator for attitudes and behaviors in the environmental domain.

Another research tradition of thinking about the future originates in research on social cognition and neuroscience. This research tradition investigates future-oriented cognitions such as mental simulation (i.e., imitative representation of some events or series of events about more proximal goals; [20]), episodic future thinking (i.e., capacity to simulate events that may come in life; [21]), affective forecasting (i.e., forecasting emotional reactions to possible future life events; [22]), and positive expectations (i.e., judging a desired future as likely; [23]). These studies mostly explored individuals’ capacity of thinking about the future and its underlying mechanisms, and mainly involved the manipulation of these cognitions and whether they influence well-being, happiness, ethical decision making and financial behavior.

The current meta-analysis draws upon Lewin’s [15] and Frank’s [16] conceptualization of FTP and the recent meta-analysis on FTP in the environmental domain [18]. Specifically, we focus on research that examined individual differences in attitudes towards the distal future and the extent to which these attitudes affect individuals’ current attitudes, decisions, and behaviors in the educational, work, and health domain.

The FTP construct differs from other motivational constructs. For example, Atkinson’s theory of achievement orientation [24] views a high instrumental value as beneficial for reaching goals in the immediate future (success or failure in the task at hand) but not for the distant future. Moreover, Malka and Covington [25] and Peetsma [26, 27] provided evidence that students’ FTP is conceptually and empirically separable from perceptions of instrumentality: FTP was a better predictor of school investment than perceived instrumentality. Similarly, FTP and delay of gratification (i.e., an ability to resist a smaller and immediate reward for the sake of the bigger but later reward) are different constructs [28] with FTP being characterized by activity rather than passivity [29]. Finally, FTP theory differs from goal setting theory [30] as this latter theory lacks the “issue of time perspective” (p. 400).

Recent research has shown that FTP influences actions in the present not only when individuals’ consider their own future but also the collective future (e.g., [31]). Referring to individuals’ FTP, Lewin [32] reasoned that an individual’s *life space* consists of geographical and social surroundings but also includes a time dimension. Lewin defined FTP as the “scope of time ahead which influences present behavior” (p. 879) and he claimed that change in FTP is one of the “most fundamental facts of development” (p. 879). Corroborating this view, Nuttin [33, 34] and Nurmi [35, 36] elaborated on the motivational force of FTP by characterizing FTP as the key “motivational space” of humans [34] (p. 63). Nuttin [33] described FTP in terms of three basic processes: motivation, planning, and evaluation. Motivation refers to people’s interests in the future, planning refers to how individuals plan the realization of their interests, and evaluation refers to the extent to which individuals expect their interests to be realized.

According to de Volder and Lens [37], FTP’s motivational force stems from two human capacities: the capacity to anticipate events and behavioral outcomes in the distant future (the cognitive component) and the capacity to ascribe valence to goals in the distant future (the dynamic component). The cognitive component reflects the instrumental value of a behavioral act, whereas the dynamic component reflects the incentive value of distant goals and the achievement of rewarding subgoals that precede the distant goal. In addition to these two components, researchers [38, 39] have added an affective-motivational component, which refers to the feelings associated with the distant future (an optimistic or pessimistic view on a particular future life domain). These instrumental, incentive, and affective values of distant goals are part (either individually or in combination) of the different FTP constructs that have been developed in the FTP literature. Across multiple disciplines (e.g., psychology, sociology, education, medicine), FTP has been generally defined as an attitude towards the future and reflecting on the future consequences of one’s present actions [40–43].

Since the middle of the 20th century, researchers (e.g., [16, 32, 44]) have posited that FTP is important for predicting various individual attitudes and behaviors in different life domains and across ages and cultures. The notion of “the future as the building site of constructive behavior and human progress” [45] (p. 40) has sparked research on the FTP construct in the central life domains of education, work, and health [2, 11, 19, 29, 33, 34, 46–49]. Education researchers have explored the relationships between FTP and educational outcomes such as learning attitudes, academic engagement, and achievement (e.g., [41, 50]). In the domain of work, researchers have linked FTP to career decision making and planning, career-choice satisfaction, and vocational maturity (e.g., [51, 52]). Researchers in the field of health have studied FTP as a possible predictor of many addictive attitudes and behaviors such as smoking, alcohol use, physical exercise, and healthy eating habits (e.g., [53, 54]). Generally, these studies indicate that individuals differ in the extent to which they think and feel about the future and in the amount of effort they put into realizing their future goals.

FTP seems to predict individual motivation, development, and behavior in the domains of education, work, and health. However, after eight decades of primary studies revealing the effects of FTP, no cumulative evidence has emerged that could (with accuracy) confirm and generalize the robustness and magnitude of FTP as a driver of motivation and behavior in these life domains. Accordingly, we unwrap four inquiries relevant to FTP as a motivational variable. First, researchers have studied the FTP construct in the separate domains of education, work, and health—with each domain utilizing their own domain-relevant operationalizations and measures. Because motivation in these life domains lies at the core of human functioning, systematically reviewing these domains together may provide substantial evidence that FTP effects hold across life domains. Thus, we ask: *How strong are the FTP effects across the education*, *work*, *and health domains?*

Second, the magnitude of the relationship between FTP and specific outcomes varies within each life domain, which further complicates the understanding of FTP as a driver of motivation and behavior [40, 55, 56]. For example, many studies in education found a significant relationship between FTP and learning outcomes (e.g., GPA; [47, 57]), whereas other studies found small or nonsignificant relationships [40, 58]. One possible reason for the disparity of research findings is that certain studies use different FTP constructs and measures, making it difficult to compare them. Some FTP constructs are based on a single FTP component such as affect (expression of affect regarding the future), cognition (ideas about the future), or behavioral intention (targeted future behavior), whereas other constructs encompass a mixture of these (e.g., [26]). Accordingly, FTP constructs consist of different FTP components which may cause the variation in FTP effect sizes. Likewise, while some FTP measures explicitly center on the life domain of education, work, or health, others exhibit a more general focus—without referring to a specific domain. Over the years, researchers have emphasized that subsequent studies should focus on the content of the FTP measure [2, 39], yet we do not know which type of FTP measure (i.e., its components and focus) is most predictive. For this reason, we ask: *Do the strengths of the relationships between FTP and outcomes depend on the type of measure and focus of the FTP construct?*

Third, another reason for the differences in effect sizes may be the cultural context of the study and/or the characteristics of the samples involved (e.g., gender, age; see [59, 60]). Because the cultural values of societies can differ significantly [61, 62], people’s time orientation (to the past, present, or future) may also differ, leading to differences in FTP effect sizes across cultures. Consequently, we ask: *Are the FTP effects generalizable across cultures?*

Fourth, the magnitude of the FTP effect size seems to vary according to the type of outcome such as individuals’ attitudes, behavioral intentions, and behaviors within a specific life domain [55, 63, 64]. For example, Gulley [55] found stronger relationships between FTP and physical activity attitudes and intention to exercise than with participation in exercise. Similarly in the work domain, Savickas et al. [64] found stronger relationships between FTP and attitudes toward vocational maturity than with decision-making ability. The question is, *does the strength of the relationship between FTP and outcomes vary among types of outcomes?*

Given the variety of FTP measures, study contexts, and study outcomes, it is important to examine whether or not existing inconsistencies in research findings may be explained or solved and whether or not FTP may be conceived of as a robust predictor of human motivation and behavior across different life domains.

### Study goals

The goal of this study was fourfold. First, we wanted to explore the relationships between FTP and outcomes in the education, work, and health domains by conducting meta-analyses on the FTP effects per life domain. Extant FTP research is scattered and lacks empirical integration within and between life domains, with respect to the magnitude of the FTP effect. Here it is worth mentioning that we did not treat the FTP domain (education, work, health) as a moderator because each life domain represented compatibility with different types of outcomes that are relevant for a specific life domain. Although reviews exist on FTP that emphasize its motivational force, these reviews were mostly conducted 10 or more years ago and primarily focused on the education domain [28, 65] and less on the work and health domains [48]. Moreover, these reviews did not seek to quantitatively summarize the empirical findings of prior FTP studies. This is achievable by means of a meta-analysis—the most powerful and accurate tool for systematically reviewing the latest research within and across research fields that have utilized disparate methods [66, 67]. Interdisciplinary meta-analyses allowed us to draw conclusions about the FTP construct as a driver of educational, work, and health outcomes.

Second, we sought to examine whether or not FTP effect sizes depend on the type and focus of the FTP measure. FTP is generally treated as a multicomponent construct [38, 39, 42], but both single and multicomponent FTP constructs exist in the literature. Due to the obvious conceptual ambiguity impeding the efforts to generate significant knowledge about the role of FTP in education, work, and health, no meta-analyses have been conducted on the relationship between FTP and outcomes in these crucial life domains. Therefore, we developed a conceptual model to review and to group the FTP constructs in order to test whether or not the type of FTP construct moderates the relationships between FTP and outcomes. We also sought to examine if the focus of the FTP measure (general vs. specific) influenced the strength of FTP–outcome relationship, so we tested FTP focus as a moderator. This ambitious goal allowed us to pinpoint which FTP construct most strongly related to educational, work, and health outcomes, thereby advancing FTP theory.

Third, we wanted to test for the moderating effects of cultural context and sample characteristics. We operationalized cultural context according to the four (out of six) cultural dimensions of Hofstede et al. [62] that were found to be related to FTP (e.g., [68]). individualism/collectivism, long-term/short-term orientation, uncertainty-avoidance, and indulgence/restraint. The last three dimensions may be of particular relevance to FTP effects as they relate to how cultures view time (i.e., short-term oriented societies are more concerned with the past and present, whereas long-term oriented societies are more concerned with the future), to how cultures approach novel situations (i.e., cultures with strong uncertainty-avoidance feel an equally strong need for a timeline and outcome expectations, whereas cultures with a low uncertainty-avoidance are more content with unknown and unpredictable situations), and to how cultures treat desire and impulse control (i.e., relatively weak control or free gratification is termed *indulgence* and relatively strong control *restraint*). Because sample characteristics (age, gender) can serve as boundary conditions of particular effect sizes, we tested them as moderators while controlling for other confounds (e.g., study design, year of publication).

Fourth, we wished to examine relationships between FTP and various outcome types. We used a seminal theoretical framework for distinguishing outcome types, namely, Ajzen and Fishbein’s [69–72] theory of planned behavior (TPB). Ajzen and Fishbein established this theory on the assumption that human behaviors are guided by attitudes toward behavior, normative beliefs, control beliefs, and behavioral intentions. The TPB helped us to integrate the findings from the different research domains and afforded us a greater theoretical and conceptual understanding of how FTP affects specific outcomes.

What follows is a summary of different FTP constructs and operationalizations. To systematically deal with the diversity of FTP constructs, we developed a conceptual model for reviewing the FTP studies across the three life domains and a general definition of FTP in order to select the FTP studies for our meta-analyses. Later on, we discuss cultural and other demographic and study variables as possible moderators of the relationships between FTP and the outcomes. With respect to grouping different outcome types, we discuss the TPB as a theoretical framework, after which we describe our method and report and discuss the results of our meta-analyses.

### FTP constructs: A conceptual model

The literature on FTP includes a variety of definitions describing it as an ability to imagine one’s future [46], the anticipation of future goals [41, 43, 73], an attitudinal concept [17, 19, 36, 42], or as an instrumental value of present activities to reach valued goals in the future [74]. Accordingly, researches have utilized different FTP conceptualizations (e.g., *future orientation*, *future focus*, *consideration of future consequences*) and measures (see [56, 75].

FTP is an attitude that encompasses personal *cognitions*, *feelings*, and *behavioral intentions with respect to the future*. Cognitions relate to thoughts about future outcomes and goals that are valued and instrumental for current decision making and behaviors (e.g., goal planning and striving). Feelings correspond to the emotions (e.g., hope and fear) that are associated with the future, and behavioral intentions relate to individual’s plans to engage in behaviors in order to realize future goals.

FTP research in different life domains has developed a variety of FTP constructs (see Table 1). For example, although some FTP constructs mainly concern individuals’ thoughts about the future [46, 56], the great majority also embraces planning or/and feelings related to the future [10, 19, 76, 77]. Other FTP constructs refer to a single component (e.g., cognition), for example, the future focus measure by Shipp et al. [56] or a mixture of cognitive, affective, and behavioral intention components (e.g., FTP on school and professional career measure, see [26, 27]).

**Table 1:** FTP constructs.

Construct	Author(s)	Definition	Conceptualization
**Future temporal depth**	Bluedorn [78]	Distance into the future that individuals and collectivities consider when contemplating events that may happen.	Ranges from short depth to long depth.
**Future orientation**	Bowels [79]	“A clear, organized approach to future events and activities” (p. 561).	Clarity and approach to future events and activities.
**FTP**	Carstensen and Lang [80]	Individuals’ perceived belief about how much time they have left in life and how they perceive it.	Open ended (perceiving future in a positive way and concentrating on the options, plans, and goals they can pursue in remaining lifetime) and limited (perceiving many restrictions and boundaries that lie in the time ahead, concentrating on losses and limitations).
**FTP**	De Volder and Lens [37]	Cognitive capacity to anticipate immediate and long-term outcomes of a task in a distant future.	Includes cognitive aspect (capacity to look far ahead in the future) and dynamic aspect (capacity to ascribe high value to long-term goals).
**Future time orientation**	Gjesme [46]	Degree to which one’s current behavior is influenced by future concerns.	Includes four components: involvement (the degree to which one focuses on future events), anticipation (how well one prepares for the future events), occupation (the amount of time one thinks about the future) and speed (the rate at which one perceives the future approaching).
**FTP**	Husman and Lens [41]	“Present anticipation of future goals” (p. 115), or as a person’s conceptualization of future and connection to it.	Two dimensions: connectedness (disposition to anticipate in the present, the long-term consequences of a potential action; it is also sometimes referred as perceived instrumentality or utility) and valence (disposition to ascribe high value to goals in the future relative to goals in the present).
**FTP**	Lewin [32]	“The scope of time ahead which influences present behavior” (p. 879).	
**FTP**	Lomranz, Shmotkin, and Katznelson [81]	“Ways in which people conceive of, organize, and feel about their future” (p. 407).	
**Future attitude**	Mello & Worrell [17]	Positive or negative attitude towards the future.	
**FTP**	Nurmi [36]	Individuals’ thoughts and attitudes toward the future.	Includes motivation (what interests people have in the future), planning (how people plan the realization of their interests), and evaluation (extent to which people expect their interests to be realized).
**FTP**	Nuttin [82]	From the subjective point of view, FTP is the area of more or less distant and dense time plans where the intentional consideration over the objects influences behavior.	
**FTP**	Peetsma [26, 42]	An attitude toward a certain life domain viewed over time.	Includes cognition (ideas or expectations with regard to the future, and of social realities), affect (an expression of feeling or affection towards a particular life domain in the future), and behavioral intention.
**Future orientation**	Savickas [83]	An attitude toward planning.	It is characterized by a sense of continuity among the past, present, and future as well as optimism and hope about the achievability of goals and denoted by a sense of relatedness across time frames.
**Future orientation**	Seginer [84]	A multidimensional process related to future in different life domains (e.g., education, work family, leisure).	Three components: motivational (value, expectance, control), cognitive representation (hopes and fears), and behavioral (exploration, commitment).
**Future focus**	Shipp, Edwards, & Lambert [56]	Attention individuals devote to thinking about future.	General thinking related to future.
**FTP**	Simons, Dewitte, & Lens [74]	The instrumental value of present activities for reaching valued goals in the future.	Four different types of instrumentality emerged from combining the FTP, goal theory, and the self-determination theory: proximal utility–external regulation (the present task is compulsory and the individual is only driven by extrinsic reasons); proximal utility–internal regulation (there is no direct relation between the present and future task, but the present activity is internally regulated because learning and performing are a goal in itself); distal utility and external regulation (future goals are strived for, but extrinsic rewards are at the center); distal utility and internal regulation (future goals are strived for and regulate present actions).
**Consideration of future consequences**	Strathman, Gleicher, Boninger, & Edwards [43]	The extent to which people consider the potential distant outcomes of their current behaviors and are influenced by those potential outcomes.	Immediate and future consequences of one’s current behavior.
**Future orientation**	Trommsdorff [39]	Complex multidimensional system.	Two types of components: cognitive and emotional or motivational. The cognitive component relates to the structure of the events projected into the future, both in terms of time extension (i.e. how far in the future those events are projected) and in terms of the content (i.e., the degree of realism of the objectives, the density of events projected into the future, and the clarity of those objectives). Affective or motivational component reflects the emotional valence of future events.
**FTP**	Zimbardo and Boyd [19]	An attitude that entails considering goal planning and achieving.	It is a general and positive tendency toward the future.

### FTP construct types

**Fig 1:**
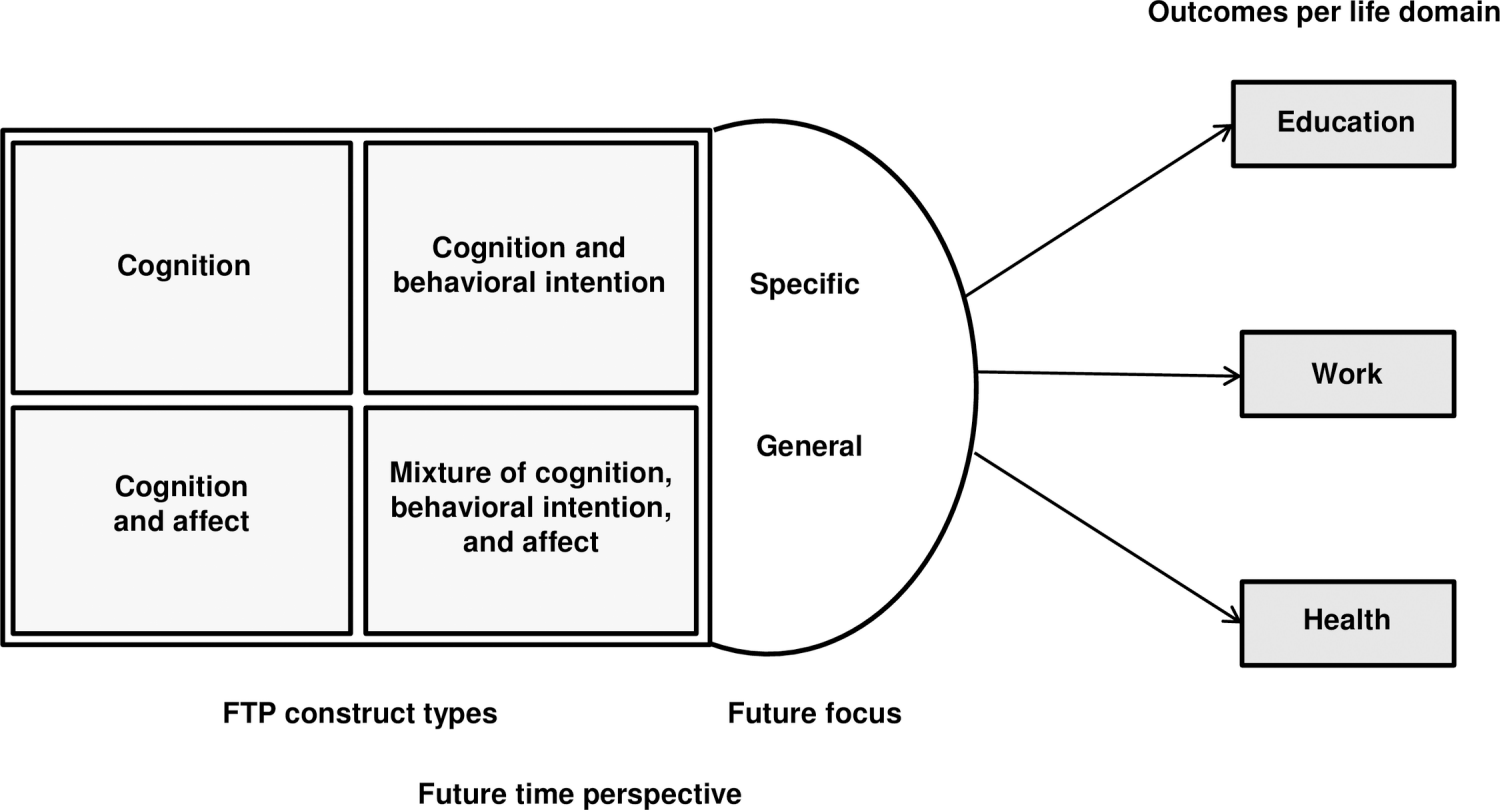
Conceptual model for grouping FTP across life domains.

In order to assemble available constructs and measures into a parsimonious model of FTP affecting motivation, attitudes, and behaviors in education, work and health we categorized these different constructs as (a) cognition, (b) the combination of cognition and behavioral intention, (c) the combination of cognition and affect, and (d) a mixture of cognition, behavioral intention, and affect (Fig 1). Cognition refers to an individual’s focus on the future (e.g., ideas). Cognition and behavioral intention together concern an individual’s thoughts, perceptions, and efforts related to the future (i.e., planning, setting future goals). Cognition and affect together concern the affective tone of future cognitions—specific emotions relate to future goals (e.g., happiness, worry, fear)—and the last category combines cognition, affect, and behavioral intention for a certain action. Based on the presence and combination of the components (i.e., cognition, behavioral intention, affect), each FTP measure can be assigned to one of our four FTP construct types.

Because affect and behavioral intention play a pivotal role in goal-directed behavior [34, 36, 39, 50, 84], we propose that FTP constructs that embrace thinking about the future, including cognition, feelings, and behavioral intentions, will be more strongly related to educational, work, and health outcomes as compared to FTP constructs that merely include cognition and/or affect.

### FTP focus

Although Nuttin and Lens [45] stated that “time perspective cannot be conceived independently of its content” (p. 23), some FTP measures are more general, meaning they do not specify the context, whereas others focus on a specific life domain. Some examples of general FTP measures include Zimbardo and Boyd’s [19] Future Time Perspective scale and Strathman, et al.’s [43] Consideration of Future Consequence scale (CFCS). The CFCS includes items such as “I believe that a person’s day should be planned ahead each morning” and “I think it is more important to perform a behavior with important distant consequences than a behavior with less important immediate consequences.”

Examples of FTP measures that focus on a specific life domain include scales developed by Peetsma [26] and Seginer, Nurmi, Poole, and Shoyer [85]. These measures specify the life domain by including items that explicitly refer to school, future career, or homework (e.g., “I like to think of my future work or study,” “I am making serious preparations for my future education”). Fong and Hall [86] developed similar measures in the health domain and included items such as “I spend a great deal of time thinking about how my present eating habits will affect my life later on” and “I never consider the long-term consequences of staying fit before I exercise.” Based on the principle of compatibility [87, 88], which requires that attitudinal and behavioral measures involve similar actions, targets, contexts, and time elements, we propose that domain-specific FTP measures will show stronger relationships with outcomes in education, work, and health than general FTP measures.

### Cultural context

Researchers have argued that an individual’s FTP depends on his or her cultural identity [2, 48, 60, 89–91]. McInerney [60] postulated that culture may influence the extension of future thinking as societies differ in values (e.g., contributing to the development of the society or preserving the status quo). Based on the time-perspective profiles of 24 countries, Sircova et al.[68, 92] found significant and strong associations between country-level FTP scores and Hofstede et al’s. [62] cultural dimensions of uncertainty-avoidance and indulgence/restraint. In our meta-analyses, we built upon these theoretical notions and prior research by exploring whether or not FTP–outcome relationships depend on the cultural context as operationalized with Hofstede’s cultural dimensions.

The individualism/collectivism dimension reflects whether people’s self-image is defined in terms of “I” or “we” [61, 93]. Although individualistic cultures prioritize personal achievements, success, and aspirations over community goals (e.g., the goals of a family or of an organization), collectivistic cultures value shared goals. It has been found that individualistic, compared to collectivistic cultures are more future oriented because they focus on abstract events and universal rules that are applicable across situations—as opposed to concrete and particular events situated in the present time [94]. This finding suggests that individuals in individualistic cultures are more concerned with their future and are better able to envision it. Consequently, we expect that FTP will relate more strongly to educational, work, and health outcomes in individualistic cultures than in collectivistic cultures.

Long-term/short-term orientation refers to how cultures view time, which directly links to the relevance of the FTP construct in a particular culture. These Hofstede dimensions and present and future scales of the Zimbardo Time Perspective Inventory represent different constructs [92, 95]. Cultures demonstrating short-term orientation are more concerned with the past and present and pursue quick results, whereas cultures demonstrating long-term orientation are more concerned with their future and pursue future-oriented goals. In a study including 93 countries, Hofstede et al. [62] found a significant association between long-term orientation and school results. Accordingly, we expect that compared with short-term oriented cultures, FTP will be more salient in long-term-oriented cultures and that the FTP–outcome relationship will be stronger in these cultures.

Uncertainty-avoidance reflects individuals’ level of comfort with unstructured and unpredictable situations [96]. Whereas cultures high in uncertainty-avoidance may feel a strong need for a definitive prognosis, timeline, and outcome expectations, cultures low in uncertainty-avoidance may be more content with the unknown and have less need for cognitive closure [62]. Consequently, we propose that FTP will more strongly relate to educational, work, and health outcomes in high compared to low uncertainty-avoidance cultures.

Indulgence/restraint involves the extent to which a society controls desires and impulses. Relatively weak control is termed *indulgence* and relatively strong control *restraint*. A society that permits “relatively free gratification of basic and natural human desires related to enjoying life and having fun” [96] (p. 15) represents indulgence, compared to a society that suppresses and controls gratification by means of social norms. Sircova et al. [92] found that cultures high in indulgence have a lower FTP than cultures low in indulgence. Based on this, we expect that the FTP–outcome relationships will be weaker in cultures with high indulgence than in cultures with low indulgence.

### Additional sample and study moderators

Demographic variables such as age and gender have been found relevant for FTP [46, 59]. For example, Gjesme [46] found that girls thought more about the future than boys. Study design and year of publication may also be relevant for the FTP–outcome relationship. Cross-sectional data may present higher correlations than longitudinal data because of common method variance [97]. With respect to the publication year, it would be interesting to explore whether or not the effect of FTP on outcomes has changed based on the year when the studies were conducted.

### FTP and different outcome types: The TPB

FTP has been related to different outcomes across the domains of education, work, and health (e.g., school performance, job satisfaction, physical activity). Grouping these outcomes in a sound framework would yield a better understanding of the FTP–outcome relationships. The TPB [71, 98] is one of the most influential and applied models used to predict and explain human behaviors in the education, work, and health domains [99–101]. The TPB relies on the assumption that human behaviors are guided by beliefs, attitudes toward the behavior, subjective norms, perceived behavioral control, and behavioral intention. Attitudes toward the behavior relate to an individual’s positive or negative evaluations of performance regarding a particular behavior and are determined by behavioral beliefs linking the behavior to different outcomes. Subjective norms refer to the perceived social pressure to perform or to abstain from a certain behavior and are determined by accessible normative beliefs regarding other people’s perception of importance. Perceived behavioral control reflects people’s perception of the ease or difficulty of performing the desired behavior; thus, it includes individuals’ beliefs in their abilities (self-efficacy) to execute a certain behavior [71]. Behavioral intentions indicate “how much effort people are planning to exert in order to perform the behavior” [98] (p. 181) and capture the motivational factors that influence that behavior.

Distinguishing the outcome types in the education, work, and health domains based on the TPB may deepen our understanding of the psychological processes involved in the FTP–outcome relationships and could explain the differences in their effect sizes. According to the TPB, behavioral intention is the most proximal predictor of actual behavior. Research supports this, but it also shows that the behavioral intention–behavior relationship is far from perfect [102]. Behavioral intentions are, in turn, influenced by attitudes, normative beliefs, and perceived behavioral control. FTP includes an individual’s beliefs about the future [10] and, depending on the specific FTP construct, encompasses cognitions, behavioral intentions, and/or affect regarding the future. Hence, FTP is conceptually related to the attitudes and behavioral intentions in the TPB, but they are not the same. FTP concerns attitudes and behavioral intentions regarding the future, whereas the study outcomes concern attitudes, behavioral intentions and behaviors in the present. However, we expect that FTP will associate more strongly with attitudinal outcomes and behavioral intentions than with actual behaviors.

## Method

### Literature search

These meta-analyses cover a period from 1984 (the earliest study) through March 2014. We used multiple techniques recommended by Lipsey and Wilson [103] and Cooper [66] in order to retrieve as many studies as possible. We created a log to keep track of the literature search (see S1 File). We searched the electronic databases relevant to the three life domains (PsycINFO, MEDLINE, ERIC, Business Source Premier, Web of Science, CINAHL, and SPORTDiscus). The main searched terms included *time perspective*, *future time perspective*, *future time orientation*, *future consequence*, *motivation*, *learning*, *achievement*, *work*, *career*, *future planning*, *decisions*, *health behavior*, and *health attitudes*. We searched for these terms in subject headings, abstracts, and in the keywords of the studies and used filters for test and measurement terms (e.g., Zimbardo Time Perspective Inventory, Temporal Focus Scale). In addition, we conducted a backward search of the reference sections of published articles to identify relevant articles missed in the computerized search (e.g., [2, 48]).

To avoid publication bias and the “file drawer problem” [104], we searched for both published and unpublished reports as well as doctoral dissertations and master’s theses via the Dissertation Abstracts Online and Google, as recommended by Johnson and Boynton [67]. In addition to the database search, we searched conference abstracts (e.g., International Conference on Life Design and Career Counseling: Building Hope and Resilience, 2013; 1st International Conference on Time Perspective, 2012) and requested emerging or unpublished significant and nonsignificant studies from prominent FTP researchers. Finally, to acquire a wide-range of responses from researchers in the time perspective field, we posted a request for published, unpublished, and emerging FTP data via the International Time Perspective Network group on LinkedIn and the Time-Research listserv.

### Inclusion and exclusion criteria

We used several criteria to select the studies. First, we required studies to fall in line with our FTP definition. We excluded studies that used the Future Time Perspective Scale (FTPS; [80, 105]) because this measure includes the amount of time that individuals believe to have left in their lives (i.e., linked to the Life-Span Theory). Also, studies that related FTP to economic markers of time perspective (e.g., delay-discount rate; [106]) were omitted. Although delay discounting and time perspective are related constructs, it is still premature to consider delay discounting and time perspective under a single construct [107]. Moreover, because direction (i.e., past or future) and distance (i.e., how far into the future) are different and generally unrelated attributes of time perspective [78], we excluded studies that used the Bluedorn Temporal Depth scale [78] and scales that did not include items explicitly relating to the future—such as the Speed and Distance (Extension) subscale from Husman and Shell’s [10] FTP measure. When FTP was measured with the Consideration of Future Consequences scale [43] and considered as a two-factor construct (i.e., immediate vs. future), we only used the future subscale because the items of the immediate subscale reflect present rather than future orientation [108, 109]. Regarding the Time Perspective scale for school and professional career by Peetsma [26], which is comprised of a short- and long-term future subscale, we excluded the first subscale because its items reflect the present time frame (e.g., “I have little use now of what I learn at school”). Studies that used the Hope scale [110] were also excluded because hope is considered a separate construct. Studies that mixed the FTP measure with other constructs (e.g., related to self-determination theory, achievement goal theory) were excluded if the reported correlation did not consider the FTP–outcome of interest per se.

Second, we required that the FTP construct be measured with a self-report method, which is dominant in FTP research and matches the psychological nature of the construct [111, 112]. We excluded studies that measured FTP with free-response measures and open-ended questionnaires. In the past (1960s and 1970s) the free-response methods were used most often (e.g., “tell me a story,” “story completion,” “TAT,” “important events,” “future events”) alongside projective methods (e.g., Cottle’s Circle tests, [113]) or a combination of free-response and scaling methods (e.g., [114]). However, all of these methods had low reliability and scoring difficulties and nowadays researchers mostly employ the scaling methods. It is important to note that we excluded studies that measured possible future work selves—a construct that relates to individuals’ self-concept and that has been mostly assessed with an open-ended measure (e.g., [115, 116]).

Third, FTP had to relate to outcomes that refer to attitudes, motivations, behavioral intentions, and behaviors in the life domains of education, work, and health. Studies that linked FTP to themes and constructs in other domains (e.g., identity formation, meaning of life, dreams, game playing) were omitted.

Fourth, we selected empirical field studies but excluded qualitative reviews and conceptual articles. After reviewing studies based on the abovementioned criteria, we found only five experimental (i.e., intervention) studies relating to educational, work, and health outcomes; only one intervention study in the work (i.e., [52]) and health (i.e., [117]) domains; and only three studies in the education domain (i.e., [118–120]). These experimental studies manipulated FTP in different ways, potentially confounding our results. Most of the interventions were related to educational outcomes; however, they were only conducted in one country (The Netherlands), involved the same type of participants, and used the same FTP measure, thus, they did not allow us to test for our moderators. Therefore, we decided to exclude these studies.

Fifth, we required studies to include a general, nonclinical sample. For example, we excluded studies with adjudicated adolescents [121] because the findings of these studies were likely confounded by specific sample characteristics, thus, were less generalizable to the general population.

**Fig 2:**
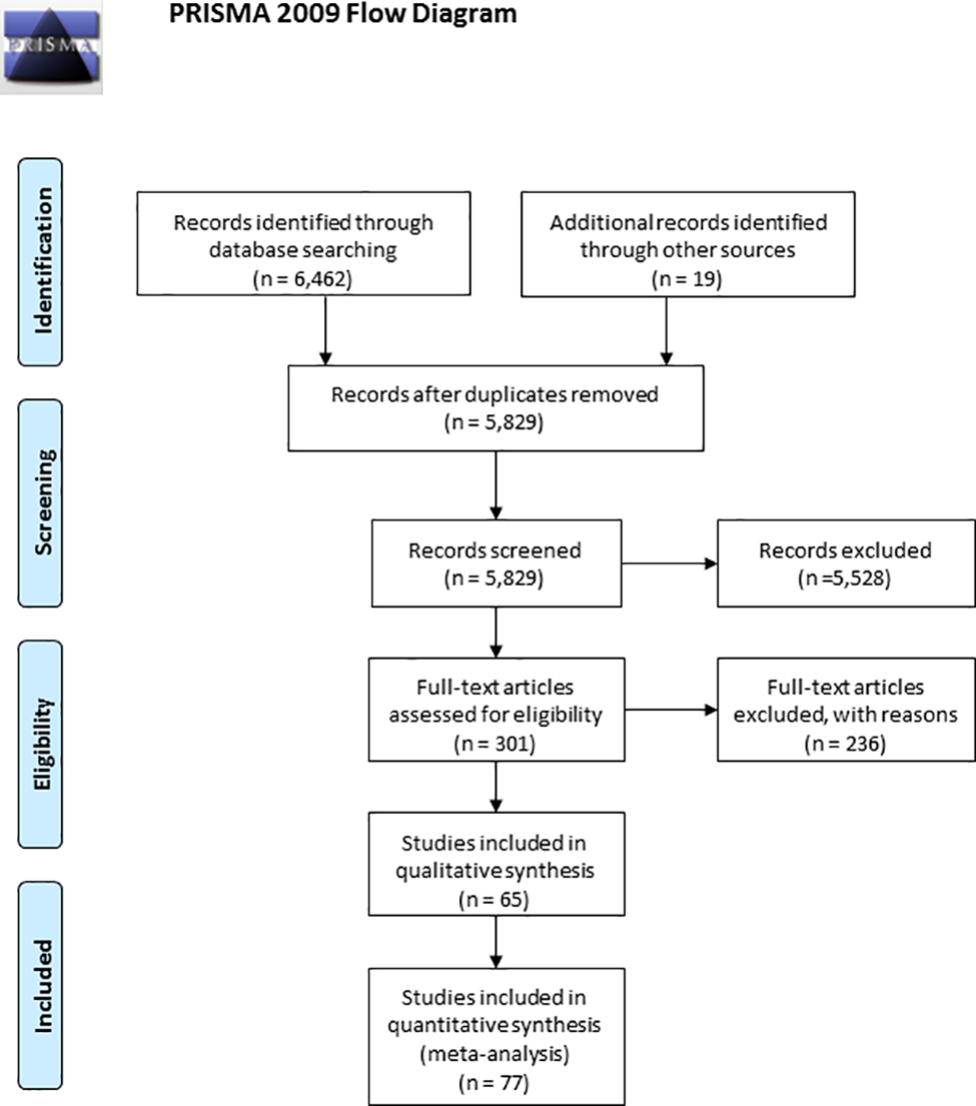
PRISMA flow diagram.

The literature search resulted in 6,481 reports. After having removed the duplicate studies (652 reports) and screened 301 potentially relevant studies, 65 reports met our inclusion criteria (Fig 2). These 65 reports included 57 published articles and 4 master or doctoral theses, two unpublished master theses and two unpublished data sets. From these 65 reports, we identified and examined 77 independent samples that met our inclusion criteria. Consequently, in the rest of the paper we refer to these independent samples as included studies (*k* = 28 in the education domain, *k* = 17 in the work domain, and *k* = 32 in the health domain). The studies were published between 1984 and 2014 and involved *N* = 31,558 participants. Samples originated from the USA (35), Western Europe (34), Australia and New Zealand (5), Asia-Pacific (2), and Eastern Europe (1). The mean age of the total sample was 22.33. On average, the sample included 46.16% male respondents. Tables 2–4 show the references of the included studies and their characteristics per life domain.

**Table 2:** Overview and characteristics of the studies in the education domain.

Study	*N*	FTP construct	FTP focus	Country	IC	LTO	UA	IR	Outcome type	Age	Gender	Study design	Effect size
**Adelabu [57]**	661	CBI	General	USA	91	26	46	68	VB	15	41.3	Cross-sectional	.12
**Andretta, Worrell, & Mello [40]**	300	CA	General	USA	91	26	46	68	VB	16	60	Cross- sectional	.05
**Barber, Munz, Bagsby, & Grawitch [122]**	255	CBI	General	USA	91	26	46	68	VB	19.6	21.4	Cross-sectional	.27
**Bowles [58]**	177	COG	General	Australia	90	21	51	71	VB	14.5	50	Cross-sectional	.08
**Bowles [79]**	228	COG	General	Australia	90	21	51	71	UB	16.5	49.6	Cross-sectional	.24
**Brown & Jones [123]**	261	CA	General	USA	91	26	46	68	ATB, VB	15.5	NA	Cross-sectional	.27
**de Bilde, Vansteenkiste, & Lens [124]**	275	CBI	General	Belgium	75	82	94	57	ATB, PBC, BI, UB	17	34.4	Cross-sectional	.38
**Eren [125]**	188	MIX	General	Turkey	37	46	85	49	VB	19.4	76.1	Longitudinal	.02
**Ferrari, Nota, & Soresi a [51]**	498	MIX	General	Italy	76	61	75	30	VB	11.9	50.2	Cross- sectional	.32
**Ferrari, Nota, & Soresi b [51]**	675	MIX	General	Italy	76	61	75	30	VB	15.8	49.8	Cross- sectional	.21
**Inocêncio, Gomes [126]**	402	CBI	General	Portugal	27	28	99	33	ATB, BI	16.7	38.3	Cross-sectional	.23
**Hau Yee [127]**	368	CBI	General	China	25	61	30	24	ATB, BI	18.5	41.6	Cross- sectional	.11
**Hilpert, Husman, Stump, Kim, Chung, & Duggan [128]**	546	CBI	General	USA	91	26	46	68	UB	21	83.3	Cross- sectional	.28
**Horstmanshof & Zimitat [129]**	347	CBI	General	Australia	90	21	51	71	ATB, UB	22	33	Cross-sectional	.39
**Levy & Earleywine [130]**	217	CBI	General	USA	91	26	46	68	PBC	20.8	29	Cross- sectional	.09
**Peetsma [42]**	606	MIX	Specific	Netherlands	80	67	53	68	UB	15.5	46.6	Cross-sectional	.35
**Peetsma, Hascher, van der Veen, & Roede a [131]**	71	MIX	Specific	Netherlands	80	67	53	68	UB	14	50	Cross-sectional	.50
**Peetsma, Hascher, van der Veen, & Roede b [131]**	78	MIX	Specific	Germany	67	83	65	40	UB	14	50	Cross-sectional	.13
**Peetsma, Hascher, van der Veen, & Roede c [131]**	204	MIX	Specific	Czech Republic	58	70	74	29	UB	14	50	Cross-sectional	.43
**Peetsma, Hascher, van der Veen, & Roede d [131]**	134	MIX	Specific	Switzerland	68	74	58	66	UB	14	50	Cross-sectional	.40
**Peetsma, Schuitema, & van der Veen [50]**	678	MIX	Specific	Netherlands	80	67	53	68	PBC	12.8	52	Longitudinal	.28
**Peetsma, & van der Veen [47]**	906	MIX	Specific	Netherlands	80	67	53	68	UB, VB	12.5	55	Longitudinal	.26
**Peters, Joireman, & Ridgway [132]**	231	MIX	General	USA	91	26	46	68	VB	19	32.5	Cross-sectional	.29
**Rodrigues Nobre [133]**	134	CBI	General	Portugal	27	28	99	33	ATB	14.7	50	Cross- sectional	.37
**Seginer & Mahajna [134]**	295	COG	Specific	Israel	54	38	81	NA	VB	17	0	Cross- sectional	−.02
**Shell & Husman [135]**	198	MIX	General	USA	91	26	46	68	UB, VB	19	30	Cross-sectional	.13
**Stachowski [136]**	94	CBI	General	USA	91	26	46	68	UB, VB	23.9	19.1	Cross- sectional	.29
**Worrell & Mello [137]**	815	CBI	General	USA	91	26	46	68	ATB, BI, VB	14.4	46.6	Cross-sectional	.20

*Note*. *N* = number of participants included in the effect size estimate; COG = cognition; CBI = cognition and behavioral intention; CA = cognition and affect; MIX = mixture of cognition, behavioral intention, and affect; IC = individualism/collectivism; LTO = long-term orientation; UA = uncertainty-avoidance; IR = indulgence/restraint; ATB = attitude toward behavior; BI = behavioral intention; PBC = perceived behavioral control; UB = unverifiable behavior; VB = verifiable behavior; NA = not available.

**Table 3:** Overview and characteristics of the studies in the work domain.

Study	*N*	FTP construct	FTP focus	Country	IC	LTO	UA	IR	Outcome type	Age	Gender	Study design	Effect size
**Eren & Tezel [138]**	423	CBI	General	Turkey	37	46	85	49	ATB, PBC, BI, UB	19.86	18.7	Cross-sectional	.21
**Eren [139]**	396	CBI	General	Turkey	37	46	85	49	BI	20.53	30.6	Cross-sectional	.34
**Ferrari, Nota, & Soresi a [51]**	498	MIX	General	Italy	76	61	75	30	ATB, PBC	11.9	50.2	Cross-sectional	.42
**Ferrari, Nota, & Soresi b [51]**	675	MIX	General	Italy	76	61	75	30	PBC, BI	15.79	48.9	Cross-sectional	.43
**Gupta, Hershley, & Gaur [140]**	236	CBI	General	India	48	51	40	26	PBC	28.14	59.7	Cross-sectional	.27
**Halvari & Thomassen [141]**	150	CBI	Specific	Norway	69	35	50	55	VB	15	44.7	Cross-sectional	−.25
**Janeiro & Marques [142]**	620	MIX	General	Portugal	76	61	99	33	ATB, UB	16.04	44.8	Cross-sectional	.25
**Rosseel [143]**	170	CBI	General	Belgium	75	82	94	57	ATB	17.5	47	Cross-sectional	.19
**Savickas, Silling, & Schwartz [64]**	97	MIX	General	UK	89	51	35	69	ATB, PBC, BI	19.5	62.9	Cross-sectional	.33
**Shipp [144]**	132	COG	General	USA	91	26	46	68	ATB, PBC, BI	38.16	NA	Cross-sectional	.16
**Shipp, Edwards, & Lambert [56]**	362	COG	General	USA	91	26	46	68	ATB, BI	39	NA	Longitudinal	.07
**Shirai, Shimomura, Kawasaki, Adachi, & Wakamatsu [145]**	3345	CA	General	Japan	46	88	92	42	ATB, PBC, BI	30.13	28	Cross-sectional	.19
**Strauss, Griffin, & Parker a [146]**	397	MIX	General	USA	91	26	46	68	ATB, UB, VB	42.67	50	Cross-sectional	.23
**Strauss, Griffin, & Parker b [146]**	103	MIX	General	USA	91	26	46	68	ATB, UB	35.68	47.1	Cross-sectional	.55
**Strauss, Griffin, & Parker c [146]**	233	MIX	General	USA	91	26	46	68	ATB, UB	29.91	30.2	Cross-sectional	.35
**Taber [147]**	195	CBI	General	USA	91	26	46	68	ATB, PBC	39.85	40.2	Cross-sectional	.09
**Walker & Tracey [148]**	218	MIX	General	USA	91	26	46	68	PBC	19.63	51	Cross-sectional	.19

*Note*. *N* = number of participants included in the effect size estimate; COG = cognition; CBI = cognition and behavioral intention; CA = cognition and affect; MIX = mixture of cognition, behavioral intention, and affect; IC = individualism/collectivism; LTO = long-term orientation; UA = uncertainty avoidance; IR = indulgence/restraint; ATB = attitude toward behavior; BI = behavioral intention; PBC = perceived behavioral control; UB = unverifiable behavior; VB = verifiable behavior; NA = not available.

**Table 4:** Overview and characteristics of the studies in the health domain.

Study	*N*	FTP construct	FTP focus	Country	IC	LTO	UA	IR	Outcome type	Age	Gender	Study design	Effect size
**Adams & Nettle [53]**	423	MIX	General	USA	91	26	46	68	UB	34.7	18.2	Cross-sectional	.28
**Adams & White [73]**	804	MIX	General	USA	91	26	46	68	VB,UB	50.5	41.4	Cross- sectional	.11
**Agnew & Loving [149]**	121	CBI	General	USA	91	26	46	68	ATB, BI, UB	19.45	100	Cross-sectional	.15
**Apostolidis, Fieulaine, Simonin, & Rolland [150]**	198	CBI	General	France	71	63	86	48	UB	21.8	50.2	Cross-sectional	.19
**Beenstock, Adams, & White [151]**	322	MIX	General	UK	89	51	35	69	UB	19.7	40.1	Cross-sectional	.32
**Björgvinsson [152]**	627	CBI	General	Canada	80	36	48	68	ATB, PBC, UB	19.7	NA	Cross-sectional	.12
**Burns & Dillon [153]**	106	MIX	General	USA	91	26	46	68	PBC, UB	21.1	32.1	Cross- sectional	.23
**Crockett, Weinman, Hankins, & Marteau [154]**	300	MIX	General	UK	89	51	35	68	BI	39	49	Cross-sectional	.18
**Daugherty & Brase [107]**	934	CBI	General	USA	91	26	46	68	PBC, BI, UB	18.99	37.3	Cross- sectional	.21
**Duangpatra, Bradley, & Glendon [155]**	607	CBI	General	USA	91	26	46	68	UB	23.4	52.6	Cross- sectional	.28
**Fieulaine & Martinez [156]**	240	CBI	General	France	71	63	86	48	UB	33.3	59.2	Cross-sectional	.29
**Gulley [55]**	185	CBI	General	USA	91	26	46	68	ATB, PBC, BI, UB	16	48.1	Cross-sectional	.10
**Hall & Epp [157]**	208	CBI	Specific	Canada	80	36	48	68	VB	45.21	24.8	Cross- sectional	.17
**Hall [72]**	357	CBI	General	Canada	80	36	48	68	UB	19	27.7	Cross-sectional	.21
**Halvari [158]**	128	CBI	Specific	Norway	69	35	50	55	VB	17.5	NA	Cross-sectional	.05
**Heckman, Wilson, & Ingersoll [159]**	1624	MIX	General	USA	91	26	46	68	BI	19	25	Cross-sectional	.30
**Hirsch [160]**	439	CBI	General	USA	91	26	46	68	UB	21.02	29	Cross-sectional	.22
**Joireman, Shaffer, Balliet, & Strathman a [63]**	119	MIX	General	USA	91	26	46	68	ATB, BI	21	59.7	Cross-sectional	.26
**Joireman, Shaffer, Balliet, & Strathman b [63]**	232	MIX	General	USA	91	26	46	68	ATB, BI	21	50.9	Cross- sectional	.16
**Keough, Zimbardo, & Boyd a [161]**	2627	CBI	General	USA	91	26	46	68	UB	21.42	45.8	Cross-sectional	.14
**Keough, Zimbardo, & Boyd b [161]**	206	CBI	General	USA	91	26	46	68	UB	23.6	35	Cross-sectional	.19
**Laghi, Liga, Baumgartner, & Baiocco [162]**	1350	CBI	General	Italy	76	61	75	30	UB	17.46	47.2	Cross-sectional	.19
**Levy & Earleywine [130]**	217	CBI	General	USA	91	26	46	68	UB	20.8	29	Cross-sectional	.13
**Mahon, Yarcheski, & Yarcheski [163]**	138	COG	General	USA	91	26	46	68	UB	13.1	42.4	Cross-sectional	.46
**McKay, Persy, & Cole [164]**	806	MIX	General	Ireland	70	24	35	65	UB	13.5	49.6	Cross-sectional	.11
**Pluck, Lee, Lauder, Fox, Spence, & Parks [165]**	50	COI	General	UK	89	51	35	69	UB	33.4		Cross- sectional	.08
**Polgar & Auslander [166]**	336	MIX	General	USA	91	26	46	68	ATB, PBC, BI, UB	16.3	51	Cross- sectional	.31
**Rise, Kovac, Kraft, & Moan[167]**	324	COI	General	Norway	69	35	50	55	ATB, PBC, BI, UB	24.6	76	Longitudinal	.09
**Rothspan & Read [168]**	376	COI	General	USA	91	26	46	68	UB	19	34.6	Cross-sectional	.20
**Strathman, Gleicher, Boninger, & Edwards [43]**	60	MIX	General	USA	91	26	46	68	ATB, UB	25		Cross-sectional	.32
**van Beek, Antonides, & Handgraaf a [169]**	165	COI	Specific	Netherlands	80	67	53	68	UB	21.29	40.6	Cross-sectional	.39
**van Beek, Antonides, & Handgraaf b [169]**	55	COI	Specific	Netherlands	80	67	53	68	UB	41.38	38.2	Cross-sectional	.25

*Note*. *N* = number of participants included in the effect size estimate; COG = cognition; CBI = cognition and behavioral intention; CA = cognition and affect; MIX = mixture of cognition, behavioral intention, and affect; IC = individualism/collectivism; LTO = long-term orientation; UA = uncertainty avoidance; IR = indulgence/restraint; ATB = attitude toward behavior; BI = behavioral intention; PBC = perceived behavioral control; UB = unverifiable behavior; VB = verifiable behavior; NA = not available.

### Coding the study characteristics

All studies were double coded by a trained research assistant and the first author. Based on the recommendations by Lipsey and Wilson [103] and Cooper [66], we developed a detailed coding manual (see S2 File). We also coded certain characteristics of each study: study design, life domain of the FTP–outcome relationship, FTP measure name, FTP construct type, FTP focus (general vs. specific), FTP subscale, FTP value (separate positive and negative subscales), number of FTP items used, outcome name and measure description, outcome type (based on the TPB; [71, 98]), country from which the sample originated, sample characteristics, and effect size (i.e., correlation coefficient). We divided the coding process into three parts to lessen the complexities of some studies. The coders met after coding each section to discuss specific coding issues. The analyses were performed on the mutually agreed data. Interrater reliabilities of moderator variables were calculated in ReCal [170] and yielded positive results (Cohen’s kappa > .97).

#### FTP construct types

We coded the FTP scales and subscales based on our developed conceptual model for grouping the FTP construct types (Fig 1) that relied on the reporting of authors in the primary studies. Accordingly, the FTP constructs were assigned to one of four construct types that measured a single or multiple components of the FTP construct: (a) cognition; (b) cognition and behavioral intention; (c) cognition and affect; and (d) the mixture of cognition, behavioral intention, and affect. Table 5 summarizes the description of FTP construct types followed by item examples.

**Table 5:** FTP construct type coding.

FTP construct types	Description	Item examples
**Cognition**	Include items about an individual’s ideas and expectations about the future.	“I think about what my future has in store”; “I imagine what tomorrow will bring for me.”
**Cognition and behavioral intention**	Include items about an individual’s future goals and ways to accomplish these goals (planning, setting, and self-control as delay of gratification).	“When I want to get something done, I make step-by-step plans and think about how to complete each step”; “I consider how things might be in the future, and try to influence those things with my day to day behavior.”
**Cognition and affect**	Include items that focus on the affective tone of future cognitions, that is, emotions that are associated with future goals (hope, worry, fear).	“If things don’t get done on time, I don’t worry about it”; “When I think about the future I feel happy.”
**Mixture of cognition, behavioral intention, and affect**	Include items that combine cognition, affect and intentions with regard to the future.	“I like to think of the way I will be able to develop my possibilities (capacities/talents) after school”; “I am willing to sacrifice my immediate happiness or well-being in order to achieve future outcomes.”

#### FTP focus

We coded the FTP measure as “general” if the measure did not specify a certain life domain or “specific” if the FTP items referred to a specific life domain (e.g., school, work, health).

#### Sample characteristics

Gender was coded as the percentage of males in the sample. The mean age of the sample was entered for age. If age was missing, we calculated the age average based on the provided grade level (in the education life domain) or averaged the age groups. Culture was coded based on Hofstede et al.’s [62] cultural value dimensions: individualism/collectivism, long-term/short-term orientation, uncertainty-avoidance, and indulgence/restraint. That is, we assigned the appropriate cultural value to the identified country of each sample. For example, we coded Japan as 46 for individualism, 88 for long-term orientation, 92 for uncertainty-avoidance, and 42 for indulgence/restraint.

#### Study design and year of publication

The study design was coded as cross-sectional versus longitudinal, and we also included the publication year of the report.

#### Outcome type

Based on the TPB, we distinguished four outcome types: (a) attitude toward behavior, (b) behavioral intention, (c) perceived behavioral control, and (d) behavior. We did not code for the subjective norm because there were no studies concerning this outcome type. Regarding the behavior outcome, we distinguished between unverifiable behavior (UB) and verifiable behavior (VB), that is, self-reported behavior that is potentially verifiable. See Table 6 for examples of outcome types per each life domain.

**Table 6:** Examples of dependent variables based on the theory of planned behavior per life domain.

Life domain	Outcome type	Example dependent variable
**Education**	ATB	Attitude toward schooling
	BI	Learning strategy
	PBC	Control beliefs about learning
	UB	Preparation for assessment
	VB	Grade point average
**Work**	ATB	Career choice satisfaction
	BI	Planned effort
	PBC	Capability beliefs
	UB	Career exploration
	VB	Weekly working hours
**Health**	ATB	Physical activity attitude
	BI	Intention to use a condom
	PBC	Self-efficacy-diet
	UB	Physical activity (daily exercise)
	VB	Body mass index

*Note*. ATB = attitude toward behavior; BI = behavioral intention; PBC = perceived behavioral control; UB = unverifiable behavior; VB = verifiable behavior.

### Meta-analytic procedures

#### Effect size estimation

Pearson’s *r* was used as the effect size index. It is the recommended effect size measure [171] and a common statistic reported by the majority of the included studies. The computations were based on reported zero-order correlations and sample size. In cases where *r* was not provided, we requested that the authors send us the correlation(s) of interest—these requests were mostly fulfilled. In a few cases, we used Spearman’s rho that was subsequently transformed to Fisher’s *z* [172]. A positive correlation indicated that a high FTP score was associated with higher outcomes in the education, work, and health life domains. When necessary, we reverse scored correlations so that the higher score represented higher, positive levels in educational, work, and health outcomes.

#### Analysis strategy

Following Hedges and Olkin [173], we performed the analyses on correlations transformed into Fisher’s *z* scores, which were then converted back to correlations. Because we assumed both systematic and random variation in the distribution of effect sizes, the obvious choice was the random-effect model. Due to power issues, however, we also reported the fixed-effect model. The random-effect model allowed us to make general inferences—which reached beyond the studies included in these meta-analyses—and to take into account both the within- and between-study errors [174], whereas the fixed-effect model allowed a more powerful test to detect significant effects. Because random-effect models are typically deemed more conservative, they can result in type II errors—an acknowledged limitation [103]. By testing both models, we applied the sensitivity analysis [175], which allowed us to examine the effects of different assumptions on the outcomes of the meta-analysis. Each effect size was weighted by the inverse of its variance. More weight was assigned to larger samples (in the fixed-effect model), whereas the weights were more balanced in the random-effects model. That is, large samples lost influence and small samples gained influence [174, 176]. Also, 95% confidence intervals were calculated for weighted average effects. If the confidence interval did not contain zero, then we rejected the null hypothesis (meaning no FTP effect).

We computed effect sizes and conducted the moderator analyses using Comprehensive Meta-Analysis software (CMA; [177]), which dealt with the complex data structures found in our meta-analyses. Correlations around .10 we considered small; around .30 as medium, and any correlation around .50 we considered as large [178].

#### Multiple effect sizes

As noted in recent reviews on meta-analysis techniques [179, 180], researchers often encounter multiple and dependent effect sizes when conducting a meta-analysis. The majority of our studies allowed us to code multiple effect sizes. This was particularly the case when coding the moderator variables and outcomes. For example, a study could include effect sizes of more than one FTP construct or outcome type. As these effect sizes are derived from the same sample, they are dependent, what violates the assumption of independence [103]. This may inflate the variance of the mean effect and may introduce a serious bias by assigning more weight to studies with more effect sizes.

According to Scammacca et al. [180], how researchers handle the effect size dependency greatly depends on the research questions addressed and the data set. Namely, are the correlations among the measures known, and are the constructs independent? We used different techniques to deal with data dependency. First, because we did not know the correlation between the outcome types in the education, work, and health domains, we averaged them with the assumed correlation for the overall analysis per each life domain. The assumed correlation was *r* = 1. According to Borenstein et al. [174], if there are more than two or three measures used in multiple studies, averaging outcomes with an assumed correlation of *r* = 1 and inflating Type II error is considered the more conservative approach. Second, in order to better understand the relationships between FTP and the different outcomes types, we ran separate meta-analyses for each outcome type.

Third, regarding the FTP construct, we applied Cooper’s [181] shifting unit of analysis technique to minimize violations of independence. This technique selects the unit of analysis and then averages the effect sizes within the unit. Based on this approach, the multiple effect sizes for FTP constructs were initially averaged to produce a single effect size for calculations involving the overall effect size for the sample. That is, we aggregated the effect sizes to produce one effect size for each study. For each moderator analysis, we aggregated effect sizes based on the particular moderator variable (e.g., the FTP construct types), such that each study only included one effect size per outcome on that particular FTP construct. This approach helped us to preserve as much of the data as possible while reducing any violations of data independency [181]. When studies included two or more subscales coded as different FTP constructs, for the purpose of moderation subgroup analyses, we were forced to code their FTP constructs according to the most dominant FTP component based on its items and averaged the subscales on that particular FTP component (i.e., [125, 134, 135, 142, 143, 148, 152]).

Fourth, if a study utilized two or more FTP measures (e.g., Zimbardo’s and a CFC scale), we selected the one that reported a higher reliability or validity [103]. Fifth, if same samples were used in multiple articles, that is, one author wrote more articles based on the same participants (e.g., [19, 57, 108]), we selected one article per each sample for inclusion based on the sample size (giving preference to the larger sizes) and the amount of available information. Also, when one article included more studies with different samples (independent studies), each study was entered separately in the analyses (e.g., [131]).

Sixth, when articles provided separate effect sizes for subgroups (e.g., boys and girls), we entered them as subgroups and used the study as the unit of analysis by merging the data as recommended by Borenstein et al. [174]. Seventh, if an outcome was measured at multiple points in time, we averaged the results for these measurements and used the average effect size in the analysis. Finally, we assigned studies that reported effect sizes for more than one life domain (e.g., GPA and health behavior) to the appropriate life domain so that a particular study contributed only one effect size to a particular life domain.

#### Moderation analyses

To test whether the dispersion of effect sizes was due to chance and sampling error or whether it reflected a real difference in effect sizes from one study to another (homogeneity test), we calculated the goodness-of-fit, *Q*, and a homogeneity index, *I*^*2*^. Significant *Q* values indicate that there are likely other variables (covariates) that explain differences in effect sizes. The *I*^*2*,^, which is based on *Q* and its degrees of freedom (*k* − 1), is the proportion of the observed dispersion due to heterogeneity. High values of *I*^*2*^ indicate more variability among the effect sizes. Values of 25%, 50%, and 75% have been suggested as reflecting low, moderate, and high levels of variance, respectively, attributable to real differences [182].

The moderation analysis addressed under what circumstances the magnitude of the effect varied. To assess the impact of our categorical moderators, we conducted fixed and random moderation subgroup analyses which are based on weighted sum of squares. For the subgroup analysis, we applied the pooled estimate of between-study variance (tau-squared, τ^2^), pooled across the subgroups, because it provided a better τ^2^ accuracy and reduced error when working with five or fewer studies per subgroup [174, 183]. Continuous moderators were examined using fixed- and random-effect meta-analytic regression. Meta-regression is analogous to regression analysis, but it uses the average effect size as outcome and study characteristics as predictors (covariates). We also found it to be a more sophisticated tool for exploring heterogeneity. Similar to regression, meta-regression yields a beta coefficient and an *R*^2^, which is the proportion of between-studies variance explained by the covariate(s). It is also analogous to the *R*^2^ index commonly reported for the proportion of variance explained by covariates in primary studies. We used a method of moments (MM) for estimating the true between-study variance, which—in contrast to other estimation methods such as unrestricted maximum likelihood (ML) and restricted maximum likelihood (REML) methods—does not depend on any assumption about the distribution of the random effects. Finally, to test the significance of moderators simultaneously, thus controlling for the other moderators, we conducted multiple regression analyses for each life domain.

#### Publication bias

In addition to including unpublished studies and dissertations to address the file drawer problem (see Method), we statistically tested publication bias using the Duval and Tweedie’s [184] trim and fill procedure (with fixed and random effects) and Egger’s test. The trim and fill method estimates the number of missing studies that may exist in a meta-analysis and is based on a funnel plot—a plot of the magnitude of the effect sizes relative to the precision of the effect sizes. If the funnel plot is asymmetrical, studies are imputed by an iterative procedure that removes the most extreme small studies from the positive side of the funnel plot and re-computes the effect size until the plot is symmetric [174]. Egger’s test assesses bias by using precision (i.e., the inverse of the standard error) to predict the standardized effect (i.e., the effect size divided by the standard error) and is considered to be a powerful test [174]. A nonsignificant value indicates a lack of bias in the data.

## Results

### Overview of the analysis

The results are reported in five steps. First, we report the results of three separate meta-analyses estimating the overall effect size for FTP on educational, work, and health outcomes. Second, we present results of publication bias tests for the three meta-analyses. Third, we explore our moderators across the three life domains. Fourth, after testing significant moderators by controlling for possible confounds, we report results of a multiple meta-regression analysis per each life domain. Fifth, we show the overall effect sizes based on the TPB in order to unpack the relationships between FTP and specific outcome types in education, work, and health. Further in the text, we report the discrepancies in random- and fixed-effect models and present the results of both analyses in the tables.

### Overall effects

#### FTP–educational outcomes

In total there were 28 independent studies (*k* = 28) concerning the relationships between FTP and educational outcomes. Table 2 shows the effect sizes along with characteristics of the included studies. We found a small-to-medium relationship between FTP and educational outcomes for the fixed-effect and random-effect model, *r* = .24, 95% CI [.20, .28], *p* < .0001. We also found a significant heterogeneity, *Q* = 128, 65, *p* < .0001. This finding is further supported by the *I*^*2*^ (*I*^*2*^ = 79.01), meaning that 79.01% of the observed variance stemmed from real differences between studies and, as such, may potentially be explained by the moderators (see Table 7).

**Table 7:** Overall effect size for FTP and educational, work and, health outcomes.

**Overall effect size for FTP and educational outcomes**														
	Effect size and 95% interval				Test of null (2-Tail)			Heterogeneity				Tau-squared		
**Model**	*k*	*r*	LL	UL	Z	*P*	*Q*	*df* (*Q*)	*p*	*I*^*2*^	T^2^	*SE*	*σ*	T
**Fixed**	28	.24	.22	.26	23.83	.00***	128.65	27.00	.00***	79.01	.01	.00	.00	.10
**Random effects**	28	.24	.20	.28	10.51	.00***								
**Overall effect size for FTP and work outcomes**														
		Effect size and 95% interval			Test of null (2-Tail)		Heterogeneity				Tau-squared			
**Model**	*k*	*r*	LL	UL	Z	*P*	*Q*	*df* (*Q*)	*p*	*I*^*2*^	T^2^	*SE*	*σ*	T
**Fixed**	17	.24	.22	.26	22.12	.00***	136.49	16.00	.00***	88.28	.02	.01	.00	.14
**Random effects**	17	.24	.17	.31	6.82	.00***								
**Overall effect size for FTP and health outcomes**														
		Effect size and 95% interval			Test of null (2-Tail)		Heterogeneity				Tau-squared			
**Model**	*k*	*r*	LL	UL	Z	*P*	*Q*	*df* (*Q*)	*p*	*I*^*2*^	T^2^	*SE*	*σ*	T
**Fixed**	32	.20	.18	.22	24.57	.00***	99.07	31.00	.00***	68.71	.01	.00	.00	.07
**Random effects**	32	.21	.18	.24	12.74	.00***								

*Note*. *k =* number of studies; *r* = effect size; LL = lower limit; UL = upper limit.

***p < .0001.

#### FTP–work outcomes

There were 17 independent samples (*k* = 17) concerning the relationships between FTP and work outcomes. Table 3 shows the effect sizes and the characteristics of the studies. The overall effect size for FTP and work outcomes was the same as in the education domain and significant for the fixed and random-effect model, *r* = .24, 95% CI [.17, .31], *p* < .0001. The significant *Q* results and the high amount of heterogeneity, *I*^*2*^ = 88.28%, again indicated the likelihood of variables moderating the overall effect (see Table 7).

#### FTP–health outcomes

There were 32 independent studies (*k* = 32) concerning the relationships between FTP and outcomes in the health domain. The effect sizes and study characteristics are displayed in Table 4. The overall effect size for FTP and health outcomes was significant and small-to-medium for the fixed and random-effect model, *r* = .20, 95% CI [.18, .24], *p* < .0001 (see Table 7). The significant *Q* results and a medium amount of heterogeneity, *I*^*2*^ = 68.71%, indicated that a significant variability was present among this sample of studies. This result warrants a further investigation of the factors to explain this variability.

### Publication bias

Using Duval and Tweedie’s [184] trim and fill approach (with fixed effects) for the relationship between FTP and educational outcomes, we found that no studies were added above the estimated average effect size. Only one study was added below the average effect size. However, the imputed point estimate was exactly the same as in the overall analysis on educational outcomes with no trimmed studies, *r* = .24, 95% CI [.22, .25]. Also, the nonsignificant Egger’s test showed that there was no bias present in this data, intercept = .23, *t*(26) = .17, *p* = .87. Under the random effect trim and fill procedure, two studies were added below the estimated average effect size, but no studies were added above the average effect size. The imputed point estimate was again the same as for the fixed-effect model, *r* = .24, 95% CI [.19, .27]. The Egger’s regression coefficient was nonsignificant and the same as for the fixed-effect, showing no bias in the data.

For the relationship between FTP and work outcomes, there were no added studies below or above the average effect size for either the fixed or random-effect trim and fill procedure. The absence of study bias was further supported by the nonsignificant Egger’s test, intercept = .41, *t*(15) = .27, *p* = .80

Regarding the relationship between FTP and health outcomes under the fixed-effect trim and fill procedure, two studies were added below the average effect size, resulting in the same effect size as in the initial fixed-effect overall analysis, *r* = .20, 95% CI [.18, .21]. Again, the Egger’s regression coefficient was not significant, intercept = .55, *t*(30) = .81, *p* = .43. The trim and fill procedure under the random-effect model added only one study above the average effect size, resulting in the same effect size as in the initial random-effect overall analysis, *r* = .21, 95% CI [.18, .24]. Taken together, these results confirmed that the three data sets did not contain publication bias for the effect size estimates and that the computed mean effect of the meta-analysis will not be biased.

### Moderator analyses

#### FTP construct type

For the relationship between FTP and educational outcomes, the FTP construct was a significant moderator in the fixed-effect model, *Q*(3) = 27.32, *p* < .001, and a marginally significant moderator in the random-effect model, *Q*(3) = 6.93, *p* = .07. The strongest relationship was found for the FTP mixture of cognition, behavioral intention, and affect (*r* = .28, *k* = 12) and for FTP cognition and behavioral intention construct (*r* = .23, *k* = 11). The weakest relationship was found for FTP cognition (*r* = .09, *k* = 3) and for the FTP cognition and affect construct (*r* = .15, *k* = 2).

We found that the relationship between FTP and work outcomes also changed as a function of the FTP construct type in both the fixed- and random-effect models, *Q*(2) = 8.39, *p* < .05 (for random model). The largest effect size was for studies that included an FTP measure that represented a mixture of cognition, behavioral intention, and affect (*r* = .34, *k* = 8). Studies that included a cognition and behavioral intention FTP construct had a smaller effect size (*r* = .15, *k* = 6). Studies with a cognition FTP construct yielded the smallest effect size (*r* = .11, *k* = 2). This was true for the analyisis with or without the one study that used the cognition and affect FTP construct (i.e., [145]).

The FTP construct significantly moderated the relationship between FTP and health outcomes in both fixed- and random-effect models, *Q*(2) = 8.89, *p* < .01 (for random-effect). All studies included either one of the FTP construct types—except for the cognition and affect FTP construct. The largest effect size was for one study that included FTP cognition (*r* = .46, *k* = 1), followed by studies that applied a mixture of cognition, behavioral intention, and affect (*r* = .23, *k* = 11), and then followed by the cognition and behavioral intention FTP construct (*r* = .19, *k* = 20). However, after removing the only study in the FTP-cognition subgroup (i.e., [163]), we found a significant moderating effect in the fixed-effect model only, *Q*(1) = 8.83, *p* < .01. Again, the stronger effect was found for studies that used an FTP measure that consisted of a mixture of cognition, behavioral intention, and affect (*r* = .23, *k* = 11), compared to studies that applied a cognition and behavioral intention FTP construct (*r* = .18, *k* = 20).

#### FTP focus

For the FTP–educational outcomes relationship, FTP focus was a significant moderator in the fixed-effect model only, *Q*(1) = 8.94, *p* < .01. FTP measures with a specific focus related more strongly to educational outcomes (*r* = .28, *k* = 8) than FTP measures with a general focus (*r* = .22, *k* = 20).

We were unable to perform the moderation analysis for the FTP–work outcomes relationships because only one study included the specific FTP measure (i.e., [141]). FTP focus did not moderate the effect size of the FTP–health outcomes relationships in both the fixed- and random-effect models. The majority of the studies included the general FTP measure (*k* = 28), and only four studies included the specific FTP.

#### Culture

The individualism/collectivism cultural dimension did not moderate the FTP–educational outcomes relationship in either the fixed- or random-effect models. However, this cultural dimension marginally predicted the FTP–work outcomes relationship in the fixed effect model, *Q*(1) = 3.16, *p* < .08, and significantly predicted the FTP–health outcomes relationship in the fixed effect model, *Q*(1) = 8.06, *p* < .005. Higher individualism went together with a stronger relationship between FTP and work and health outcomes.

The long-term/short-term cultural dimension was a significant moderator only for the FTP–educational outcomes relationship and in the random-effect model, *Q*(1) = 18.06, *p* < .001. This result indicates that the relationship between FTP and educational outcomes was stronger in countries with a long-term orientation.

The uncertainty-avoidance cultural dimension was a marginally significant predictor of the FTP–educational outcomes relationship but only in the fixed-effect meta-regression, *Q*(1) = 2.78, *p* < .09. The indulgence/restraint cultural dimension only significantly predicted the effect size of the FTP–work outcomes relationship in the fixed-effect model, *Q*(1) = 18.78, *p* < .001. Greater indulgence was associated with a weaker relationship between FTP and work outcomes.

#### Age

Age was not a significant predictor in either the fixed or random-effect meta-regressions testing the FTP–educational outcomes relationship. However, age significantly moderated the relationship between FTP and work outcomes but only in the fixed-effect model, B = −.01, *p* < .001, *k* = 17. This result indicates that the older the sample of participants were, the weaker the relationships between FTP and working outcomes would be. In testing the FTP–health outcomes relationship, age did not predict the overall effect sizes in either fixed- or random-effect models.

#### Gender

We found a marginally significant effect of gender in the fixed-effect meta-regression in testing the FTP–educational outcomes relationship, B = .001, *p* = 06, *k* = 27. This result indicates that the relationship between FTP and educational outcomes was stronger when the sample was comprised of a higher percentage of males. However, the magnitude of the slope was very small. One study had missing data on gender (i.e., [123]).

Gender significantly predicted the effect size in the fixed-effect model testing the FTP–work outcomes relationship, B = .001, *p* < .001, *k* = 15. The relationship between FTP and working outcomes was stronger when the sample was comprised of a higher percentage of males; however, the magnitude of the slope was very small, and two studies had missing data on gender (i.e., [56, 144]).

A significant moderating effect of gender was also present for the relationship between FTP and health outcomes in the fixed-effect model, B = −.001, *p* < .001, *k* = 28. Although the magnitude of the slope was very small, this result indicates that the FTP–health outcomes relationship was weaker when the sample was comprised of a higher percentage of males. It is important to note that four studies had missing data on gender (i.e., [43, 152, 158, 165]).

#### Study design

There was no difference in effect size between cross-sectional (*k* = 25) and longitudinal studies (*k* = 3) in the education domain in both the fixed- and random-effect models, *Q*(1) = .04, *p* = .85. Because the data consisted of only one longitudinal study in both the work domain (i.e., [56]) and in the health domain (i.e., [167]), we could not test study design as a moderator in these domains.

#### Publication year

Regressing the relationship between FTP and educational outcomes onto the year when a study was published yielded a null effect in both fixed- and random-effect models. The meta-regression analysis in the work domain showed no evidence that the effect size changed across years. However, publication year moderated the relationship between FTP and health outcomes in the fixed-effect model, *Q*(1) = 8.46, *p* < .005. This result indicates that the strength of the relationships between FTP and health outcomes increased over time.

### Multiple meta-regression models

#### FTP–educational outcomes

After including all significant moderators (FTP construct type, FTP focus, long-term orientation, uncertainty-avoidance, and gender) in the regression model as we tested the overall effect size of FTP on educational outcomes, we found a significant fixed-effect model. FTP construct type and FTP focus remained significant moderators, but FTP focus was only marginally significant, whereas long-term orientation, uncertainty-avoidance, and gender were no longer significant moderators. This analysis was based on 27 studies; one study did not provide information on gender (i.e., [123]).

#### FTP–work outcomes

A multiple regression analysis with FTP construct type, individualism/collectivism, indulgence/restraint, and age in the model yielded both significant fixed- and random-effect models. Due to collinearity among the covariates, we could not preform multiple regression analysis that included the gender of sample in the model [174]. In the fixed-effect model, FTP construct type and individualism/collectivism remained significant moderators. The FTP construct remained the only significant moderator in the random-effect model, explaining 11% of the variability in overall effect size, *R*^2^ = .11, *p* < .05.

#### FTP–health outcomes

When we included FTP construct type, individualism/collectivism, gender, and year of publication in the model, we found that both the fixed- and random-effect models were significant. In the fixed-effect model, all covariates remained significant moderators, but in the random-effect model, only FTP construct type remained a significant moderator and explained 30% of the variability in the overall effect size, *R*^2^ = .30, *p* < .05. This result is based on *k* = 28 because four studies had missing data on gender (i.e., [43, 152, 158, 165]).

### FTP and outcome types relationships

In the education domain, most of the studies included behavioral outcome types (*k* = 12 for FTP and UB, and *k* = 14 for FTP and VB relationships). The educational behaviors mainly concerned students’ GPAs or learning engagement such as investment in learning or study effort. Fewer studies were related to FTP and attitudes toward behavior relationships (*k* = 7), FTP and behavioral intention (*k* = 4), and FTP and perceived behavioral control (*k* = 3). The overall effect sizes for FTP and educational outcome types were significant, positive, and small-to-medium (*r* = .29 for attitudes toward behavior, *r* = .28 for behavioral intention, and *r* = .25 for perceived behavioral control). The strongest effect (*r* = .33) was for UB; the smallest effect (*r* = .16) was for VB.

In the work domain, most of the studies included correlations between FTP and work attitudes (*k* = 12), followed by studies examining FTP and perceived behavioral control (*k* = 9) and FTP and behavioral intention (*k* = 7), and fewer studies included relationships between FTP and UB (*k* = 5), and FTP and VB (*k* = 2). After conducting five separate meta-analyses on FTP and outcome type, we found a medium-to-large effect size for the relationship between FTP and UB, *r* = .40, *k* = 5, and for the relationship between FTP and perceived behavioral control, *r* = .32, *k* = 9. We found the smallest effect size in the relationship between FTP and attitudes toward work behaviors, *r* = .21, *k* = 12. Notably, we found two nonsignificant overall effect sizes: FTP and behavioral intention (*k* = 7) and FTP and VB (*k* = 2). Because the FTP and the VB relationship included only two studies, resulting in reduced power, this result, as well as the other results that were based on a small sample of studies, should be taken cautiously.

Examining the separate relationships between FTP and outcome types in the health domain, we found significant and small-to-medium effect sizes for most of the relationships in both random- and fixed-effect models: FTP and attitudes toward behavior (*r* = .14, *k* = 8), FTP and behavioral intention (*r* = .21, *k* = 9), and FTP and perceived behavioral control (*r* = .17, *k* = 6). Again, we found the largest effect size in the FTP and UB relationship (*r* = .21, *k* = 26) and a smaller effect size in the FTP and VB relationship (*r* = .14); however, this result was only based on three studies.

## Discussion

In these meta-analyses we addressed the central question whether people’s present motivation, intentions, and behaviors in the education, work, and health domains are affected by their FTP. Although research on the effects of short-term and proximal goals on human motivation and behavior [3, 5, 185] has provided rather clear results, research on the effects of attitudes toward the future has produced inconclusive findings. This comprehensive review is the first, to date, to systematically incorporate the FTP–outcome relationships found in different research fields (education, work, and health). After reviewing 77 independent studies, our key finding is that people’s FTP relates to their present motivation, intentions, and behaviors across the life domains of education, work, and health. More important, this study shows that the magnitudes of the FTP–outcome relationships are comparable across life domains (our first research question). This meta-analytic study also demonstrates that the variability in the FTP–outcome relationships can be explained by the type and focus of FTP measures (our second research question), cultural context, and some characteristics of samples and studies (our third research question). Finally, the strength of the FTP–outcome relationship seems to vary with outcome type (our fourth research question). Altogether, our findings bolster the idea that FTP is an important psychological construct for human motivation and behavior in crucial life domains and across cultures. Below, we discuss each finding and the theoretical implications, and we highlight the study’s limitations and directions for future research.

### FTP relationships with educational, work, and health outcomes

We conducted three separate meta-analyses per life domain to scrutinize the strength of the relationships between FTP and educational, work, and health outcomes. Our analyses of 28, 17, and 32 studies in the education, work, and health domain, respectively, reveal a significant, small-to-medium association between FTP and educational, work, and health outcomes for random-effect models, *r* = .24, *r* = .24, *r* = .21. These findings indicate that individuals’ cognitions, feelings, and behavioral intentions regarding their future significantly affect their educational, work, and health attitudes and behaviors. These findings also indicate that the FTP–outcome relationships are consistent across three crucial life domains, implying that the effects of FTP are not restricted to specific domains but can be generalized to a broad spectrum of situations. Indeed, a meta-analysis on FTP relationships with proenvironmental attitudes and behaviors resulted in a similar effect size as reported in our meta-analyses and highlighted the important role of FTP in the environmental domain [18]. In conjunction, these findings provide strong empirical support for the motivational role of FTP in multiple life domains.

Our findings also indicate higher variability among the FTP–outcome relationships in the education and work domains compared to the health domain. In this study, we tested whether this variability was due to the FTP measure and/or due to the characteristics of the study and samples.

#### FTP construct type

In order to explore whether the variability in the FTP–outcome relationships can be explained by the FTP measure, we used a conceptual model to group the various FTP measures into four FTP construct types: (a) cognition, (b) cognition and behavioral intention, (c) cognition and affect, and (d) a mixture of cognition, behavioral intention, and affect.

Consistent with FTP researchers who advocate that affect and behavioral intention play a pivotal role in goal-directed behavior (e.g., [34, 36, 39, 50, 84]), our results show that FTP constructs including people’s cognition, feelings, and behavioral intentions towards the future are more strongly related to outcomes than FTP constructs including cognition and/or affect only. More important, we found that the FTP construct type remained a significant moderator of the FTP–outcome relationships in the three life domains even after controlling for FTP focus (in the education domain), cultural context, and the characteristics of the samples and the study (in the work and health domains).

Overall, our findings highlight that the way in which individuals contemplate about their more distant future matters. Although the associations between FTP and outcomes were significant for each FTP construct type, they were stronger when individuals’ thinking about the future involved cognitions, affect, and behavioral intention. Based on these findings we recommend that researchers use these more comprehensive FTP measures when relevant.

#### FTP focus

We also examined whether FTP focus (general vs. domain specific) would affect the FTP–outcome relationship. Based on the principle of compatibility [87, 88]—that states that attitudinal and behavioral measures should correspond to each other at the same level of specificity (or generality) in terms of actions, targets, and contexts—we expected that the FTP measures that specify the life domain (education, work, or health) would display stronger relationships with educational, work, and health outcomes than general FTP measures. Indeed, we found that specific and general FTP measures exerted different moderating effects on the relationships between FTP and educational outcomes. That is, specific FTP measures that captured the education domain of future thinking had stronger relationships with educational outcomes than general FTP measures (without specifying the life domain). This finding corroborates extant research showing that more specific expectations and intention are better predictors than less specific expectations and intentions (e.g., [186–188]), and that more specific goals predict more effort and success in goal attainment than more vague goals (e.g., [189]). Indeed, our meta-analysis in the education domain suggests that attitudes about one’s educational future (e.g., which university to attend) promote educational-related outcomes (e.g., doing homework) more than attitudes about one’s future in general.

The distinction between specific and general thoughts about the future seems less important for health outcomes. We should note, however, that most health studies in our meta-analyses applied a general FTP measure (*k* = 28), whereas only very few studies (*k* = 4) applied a specific FTP measure. Our findings did not allow us to draw conclusions about the moderating role of FTP focus in the work domain because only one study in this domain included the specific FTP measure.

#### Cultural context

To explore the generalizability of the FTP–outcome relationships across cultures, we examined a possible moderating role of cultural context by means of Hofstede’s [96] cultural dimensions: individualism/collectivism, long-term/short-term orientation, uncertainty-avoidance, and indulgence/restraint.

In addition to Shirai and Beresneviciene [94], who suggested that individualistic cultures would have higher FTP than collectivistic cultures, we found that the relationships between FTP and work and health outcomes were stronger in more individualistic countries. This result implies that individuals in countries that value personal goals over shared goals may not only contemplate more about the future but also that they connect this contemplation more strongly to their attitudes and behaviors. In individualistic countries (e.g., USA), individuals are expected to take care of themselves and to be responsible for the realization of their goals. In order to prepare a better foundation for a secure and healthy future, they tend to be concerned with accomplishing their daily work and health goals. In contrast, individuals in countries that have a lower score on individualism (e.g., Japan) expect their relatives or members of a particular in-group to look after them in the future. For example, they may put less personal effort into attaining the benefits of tomorrow.

Cultural context did not moderate the relationships between FTP and educational outcomes. We speculate that individuals regard themselves as accountable for their own educational accomplishments in both individualistic and collectivistic cultures because these accomplishments are associated with personal aptitudes and effort across cultures [190]. For example, the amount of time students spend on their homework is a reliable and often-used measure of investment in learning and schooling across countries [131]. A recent OECD report [191] showed that homework was an indicator of PISA test scores of individuals and schools across countries. Students from collectivistic cultures, such as Japan, Hong Kong, Singapore, and Shanghai, earned the highest math scores in 2012 and also increased their score by 17 points or more per extra hour of homework [191]. The high regard for education and personal effort, combined with a need for continuous self-improvement, is embedded in the Confucianism-based view of these Asian cultures [192]. Unfortunately, this focus on educational and personal achievement may also account for the high rates of suicide amongst students who fail to perform [193].

Regarding cultural differences in the temporal orientation, we found that FTP and outcomes were more strongly associated in countries with a long-term orientation. However, this result only appeared in the education domain and not in the work and health domains. A possible explanation for this finding may be the dominance of the *short-term unattractive behaviors* (e.g., studying for an exam), but *long-term desirable outcomes* (receiving an educational degree) in the education domain, and *short-term attractive behaviors* (e.g., procrastination, smoking), but *long-term undesirable outcomes* (e.g., not finding a job, health problems), present mainly in the work and health domains. These outcomes seem to initiate different self-control systems, namely, stop and start control [194]. Stop control aims at preventing a long-term undesirable outcome (e.g., quit smoking to prevent lung cancer), whereas start control aims at attaining a long-term desirable outcome (e.g., start learning to pass an exam). Because most of the studies in the education domain included outcomes that related to start control, our findings may suggest that a long-term orientation strengthens the FTP–outcome relationship—particularly when the future outcome requires the adoption of beneficial behaviors. Temporal orientation has less of an impact on the FTP–outcome relationship when the future outcome requires the cessation of harmful behaviors.

As expected, high uncertainty-avoidance cultures displayed a stronger association between FTP and educational outcomes, yet this was not the case for work and health outcomes in our study. It is possible that individuals feel that they can control their educational achievements more so than their work and health achievements. Indeed, a recent study in the education domain [195] showed that the relationship between FTP and academic achievement was fully mediated by identified self-regulation and partly mediated by internal self-regulation. Regarding work and health, individuals may recognize specific life circumstances that are not under their control—economic conditions and available job opportunities may hinder their career; genetic susceptibility and unforeseen accidents may harm their health.

Our study indicates that the motivational strength of FTP is higher for accomplishing educational outcomes in cultures where individuals are uncomfortable with novel and unknown situations. Individuals in these cultures may view educational achievement as a way to avoid negative outcomes in the future, such as a low income and associated adverse life conditions. This finding challenges the assumption that the future is less motivating for individuals seeking to achieve educational goals in countries experiencing great political or economic uncertainty [91]. Instead, uncertain conditions may motivate individuals with developed FTP to put extra effort into their education in order to augment their chances for a better future. Moreover, it may be that individuals consider their education to be the first step to dealing with uncertainties in other life domains as well.

Building on recent results by Sircova et al. [92], we found that countries higher in indulgence exhibited a weaker relationship between FTP and work outcomes than countries lower in indulgence. This result means that individuals who experience less societal control over their desires and impulses are also less motivated by thinking about their future work than individuals who experience more societal control. As proposed, this effect may be due to the higher importance of free gratification of needs and desires (e.g., leisure activities) in cultures that promote indulgence [96]. The significant moderation effect of the indulgence/restrained dimension can also be linked to the positive and strong association between delay of gratification and FTP (e.g., [28, 196]). Students who are able to delay gratification are more likely to believe that performing well in exams will help them to attain a better future career than their impatient and pleasure-seeking counterparts [196]. Likewise, a recent longitudinal study by Watson and Milfont [197] provided evidence that change in students’ consideration of future consequences is positively related to their ability to give preference to larger versus shorter delayed rewards.

#### Additional sample and study moderators

In our findings, age played a significant moderating role in the relationship between FTP and work outcomes. That is, FTP was less motivating for work-related attitudes and behaviors as people age. An explanation for this finding can be derived from socioemotional selectivity theory [198]. This life-span motivation theory focuses on the types of goals that motivate individuals (i.e., knowledge- and emotion-related goals). The theory claims that as time horizons shrink with age, individuals become increasingly selective regarding their goals and pursuits and, instead of focusing on knowledge-related goals (e.g., career planning, knowledge acquisition), focus more on emotion-related goals (e.g., emotionally gratifying interactions). This shift in focus is proposed to be due to the age-associated shift in time perspective—from open-ended to constrained. Although an open-ended time perspective relates to prioritizing goals aimed at knowledge acquisition and the pursuit of advancement, a constrained-time perspective relates to emphasizing short-term goals that optimize well-being [4]. The FTP of older individuals is likely to be more constrained than the FTP of younger individuals; the consequence thereof being that older individuals shift their priority from work-related to emotion-related goals.

In addition to age effects, we found gender differences in the degree of strength in FTP–outcome relationships, which corroborate extant research [46, 55, 77]. Our findings suggest that contemplating the future motivates men more for the educational and work outcomes, whereas for women, thinking about the future motivates them more for the health outcomes. These findings can be explained by traditional gender-based stereotypes of the different societal roles that men and women adopt [199]. That is, men are expected to secure an existence for their families and to be devoted to their work and career, whereas women are seen as caregivers, thus, more concerned with their health for their family’s sake. Indeed, women rated higher on health-related FTP and seemed to put more effort in their health-related outcomes (e.g., be more physically active, pursue healthy eating habits) than men [55].

The other study characteristics that we tested as possible moderators of the FTP–outcome relationships concerned study design (cross-sectional vs. longitudinal) and the year of publication. Study design could not be tested in the work and health domains due to a lack of longitudinal studies in these domains. However, the design of the study did not moderate the relationships between FTP and educational outcomes.

The year of publication emerged only as a significant moderator of the relationships between FTP and health outcomes—the strength of these relationships increased over the years. This finding can be explained by the fact that more recent studies [63, 164] applied an FTP measure that included a mixture of cognition, behavioral intention, and affect—an FTP construct type that yields the strongest effect sizes. It is important to note that significant moderating effects should be interpreted with caution because the findings cannot be generalized beyond the samples included in our meta-analyses.

#### FTP and outcome types relationships

The final aim of our review was to identify whether the strength of the relationships between FTP and outcomes varies with outcome types as based on the TPB (i.e., attitude toward behavior, behavioral intention, perceived behavioral control, and unverifiable or self-reported and VB). For each of the five outcome types we conducted additional meta-analyses per life domain. We anticipated that FTP would have stronger associations with attitudinal outcomes and behavioral intentions than with actual behaviors. From our findings emerged a novel insight into the observed relationships and revealed different patterns of FTP associations relative to the outcome type.

As expected, FTP was more strongly related to educational, work, and health attitudes and to educational and health behavioral intention than to behaviors in these life domains. However, this was only true when VBs were taken into account (e.g., students’ GPA, physical activity assessed with an accelerometer). This finding that measures of FTP had a stronger relationship with social cognitions than with measures of actual behavior, is consistent with research on the gap between intentions and behavior [200, 201]. When individuals’ educational, work, and health behaviors were assessed with self-reports, FTP showed medium-to-large associations with educational and work behaviors and small-to-medium associations with health behaviors.

It is important to acknowledge that we found two nonsignificant associations in the work domain between FTP and behavioral intention as well as FTP and VB. The first nonsignificant association may be due to one large sample that showed a negative association (i.e., between FTP and job-search intention)—this had a strong influence on estimating the overall effect size. After removing this large sample, we found a significant small-to-medium association between FTP and behavioral intention. The second nonsignificant association was only based on two studies.

Although we did not anticipate strong associations between FTP and perceived behavioral control, our meta-analyses revealed robust and generalizable relationships across the three life domains. We found medium-to-large associations between FTP and perceived behavioral control in education and work and small-to-medium associations between FTP and perceived behavioral control in the context of health. These findings suggest that individuals engage in reflecting about their future to the extent that they perceive their actions to be under their own control. For example, students who think more about their futures may feel more capable about their ability to do homework or to prepare for an exam than students who involve themselves less in reflections about their futures. In the health domain we also found significant and positive relationships between FTP and perceptions of behavioral control, but these relationships were small-to-medium. Individuals who engage in thinking about future may have faith in their ability to engage in health-related behaviors (e.g., physical activity, quit smoking).

Our notion that individuals particularly reflect on future that they believe is controllable is supported by recent studies [202, 203], which found strong relationships between FTP and general and specific self-efficacy, that is, “people’s beliefs about their capabilities to exercise control over their own level of functioning and over events that affect their lives” [204] **(**p. 257). It seems that individuals who feel capable, and who reflect on their future, also see the importance of their future achievements and consequently work harder to develop the skills needed to achieve their goals. In this way, they develop realistic aspirations and select tasks they feel they can master.

### Theoretical contribution

After eight decades of FTP research, powerful evidence of the aggregated research efforts is required to appraise and lift FTP theory to the next level. Our meta-analyses contribute to current theory on FTP as a driver of human motivation and behavior in several ways. First, our findings synthesize research on the relationships between FTP and outcomes from different disciplines of psychology: education, work, and health. To date, the overall relationships between FTP and outcomes and their possible moderators were unknown within each of the three disciplines. Moreover, although there was meta-analytic evidence on the relationship between FTP and attitudes and behavior within the environmental domain [18], there was no evidence that the FTP–outcome relationships would hold or would be comparable across multiple life domains. Our three meta-analyses confirmed that FTP, besides being related to proenvironmental attitudes and behaviors, also significantly relates to individuals’ attitudes and behaviors in the life domains of education, work, and health and that the magnitude and importance of these relationships is generalizable across these life domains. Thinking about the future motivates individuals to pursue activities that help them attain distant goals.

Second, we developed a framework for grouping the FTP construct types and distinguished specific from general FTP measures in order to synthesize contradictory research findings across the life domains [48, 205]. Our results provide important implications for FTP theory as they showed that FTP measures that include cognition, affect, and behavioral intention are more strongly related to attitudes and behaviors than FTP measures that only include cognition and/or behavioral intention. Also, as evidenced in the education domain, specific FTP is more strongly related to attitudes and behaviors than general FTP.

The finding that FTP construct type in the three life domains and FTP focus in the education domain can explain variation in the FTP–outcome relationships solves, to some extent, the present inconsistencies in FTP research. Furthermore, this finding contributes to the discussion about the operationalization and measurement of FTP—a recognized obstacle in FTP research. Researchers in the education domain have stressed the importance of a comprehensive FTP measure [38, 39]. Our study provides robust evidence that it is indeed important to simultaneously consider individuals’ thoughts about the future as well as their affect and behavioral intention as predictors of educational, work, and health outcomes. In this way, we extend the theoretical framework of de Volder and Lens [37] by demonstrating that the affective–motivational component (i.e., feelings associated with the distant goals) is, in addition to the cognitive and dynamic components, a crucial motivational force of FTP. Affect and emotions form the basis for all rational computation and guide individuals’ goals and decision-making [206]. Recent behavioral and neurocognitive research has revealed that individuals’ thoughts about the future, in particular, evoke affective responses. That is, individuals experience more intense emotions when they anticipate future experiences (actual or hypothetical) than when they contemplate past experiences [207]. Furthermore, positive thoughts are more frequent and more specific and are more associated with visual images than negative thoughts [208], suggesting that it is the valence of thinking about the future that determines its motivational force.

A third contribution of our study is that we tested cultural context as a factor that could influence the strength of FTP effects. Researchers often state that findings should be explored in a generalized manner, taking different research contexts into account [209]. Extant FTP research has been conducted across different countries and continents, but the question of whether cultural context influences studies’ findings remained unsolved. In this study, we found that cultural context can weaken or strengthen the FTP–outcome relationships. This means that the results of FTP research should be interpreted in the context of the culture in which the data were collected, and interventions used to enhance FTP should be tailored to a specific cultural context. Our findings have implications for FTP theory because this theory could include culture as a significant moderator of FTP–outcome relationships. Our findings call for more cross-cultural research to further explore the specific processes underlying the moderating role of culture.

Fourth, we added to FTP theory and research by identifying age and gender as significant moderators. Both the content of FTP and the strength of its effect seem associated with age; therefore, FTP theory could incorporate age as a significant variable and could develop propositions about its specific role. FTP and age are, by definition, time-related constructs that cannot be studied independently. Future studies could, for example, investigate how the content of the FTP construct develops over people’s lives. In addition, the identified gender differences in the three life domains signal that gender should be controlled for in future FTP research. FTP theory should also adopt the notion that FTP effects depend on individuals’ stereotypical gender roles and expectations.

Fifth, we used the TPB as the theoretical framework to distinguish FTP outcomes across different research domains. Our results stress the need to better integrate seminal theories on motivation and self-regulation into FTP theory. We found that FTP exhibits the strongest relationships with attitudes across domains and with behavioral intention in the education and health domains; we found the weakest relationships to be between FTP and actual (verified) behaviors in the three life domains. However, the week relationships between FTP and actual behaviors, versus strong relationships between FTP and other (non-verifiable) outcome types might be–at least partly–due to the overlapping nature of measures based on its content and/or methodology (i.e., common method variance [97]). Nevertheless, the magnitude of the FTP and actual behaviors relationships, albeit small, offers a substantial support for FTP theory, and more studies should include measures of actual behaviors as influenced by FTP. Perceived behavioral control, a key variable in the TPB, appears to be significantly associated with FTP: Individuals reflect on future plans that (they believe) they are capable of controlling. In general, more work can be done on depicting and explaining the self-regulation processes in FTP theory [74, 195].

### Limitations and future directions

Although the current study provides the first synthesis of research on the relationship between FTP and educational, work, and health outcomes, it is not without limitations. Below, we highlight the limitations of our study and offer directions for future research in order to guide researchers and to advance the FTP field.

#### Methodical considerations

First, because the studies included in our meta-analyses used correlational designs, causal inferences cannot be made for the examined relationships. Although the findings of the included studies provide guidelines for further theory development, research, and interventions in the education, work, and health domain, we recommend that future studies explore the causal effects of FTP with longitudinal study designs and intervention studies. For example, a longitudinal study by Chua, Milfont, and Jose [210] explored long-term relationships between adolescents’ FTP and a change in well-being (e.g., happiness, vitality and sleep) via the use of coping strategies.

Second, future studies should be careful when using and interpreting FTP and outcome measures that include content overlap (i.e., specific FTP measures) or apply the same methodology (i.e., self-reports), which was the case in some of the included studies in our meta-analyses. In survey data, specific FTP measures (i.e., “I have a good sense of how I can keep fit throughout my life span”) and self-reported measures of behavioral intention (i.e., “I intend to practice physical activities next week”) may, despite their different orientation (the future vs. the present), correlate at least partly because of common method variance that tend to inflate the effect sizes [97]. Similarly, although the TPB [98] is a widely accepted and robust framework linking motivational concepts (attitudes, intentions) and behaviors and that offered us a meaningful way to distinguish different outcome types, a certain amount of content overlap may exist between some FTP measures and the categorizations of outcomes based on the TBP. To address possible bias due to common method variance, future studies could use more objective behavioral measures when examining the relationships between FTP and outcomes.

Third, because some subgroup analyses were based on five or fewer independent correlations, the generalizability of these findings is restricted. This calls for more studies to be conducted—particularly in the domain of work. Fourth, regression analyses examining the moderation effects required us to synthesize correlation matrices despite missing data in a few cases; however, this was only in the case of gender.

### FTP measure

Because the aim of our study was to synthesize FTP research in three important life domains, we focused on the FTP conceptualization that was most often studied in these domains (i.e., FTP as an attitude that encompasses personal *cognitions*, *feelings*, and *behavioral intentions with respect to the future*) and we used a conceptual model for grouping the different FTP measures within this research tradition. This meant that some FTP measures and subscales were omitted (e.g., FTP measure by Carstensen & Lang [80]; Speed and Distance subscales by Husman & Shell [10]). Also, we focused on more realistic future thoughts as drivers for motivation, but some forms of future thinking may be less beneficial for motivation. For example, when people focus on the future in an overly idealistic way this actually prevents them from mobilizing effort and in this way may dampen motivation and goal-directed behavior [211, 212]. However, constructs such as positive fantasies did not align with our FTP conceptualization, nevertheless, we acknowledge them and suggest that future researchers explore their overall effects on outcomes in education, work, and health and test their relationship with the FTP conceptualization used in our study. Relying on our parsimonious framework that integrated diverse FTP measures, we suggest that future studies (a) incorporate FTP measures that relate to cognition, affect, and behavioral intention, (b) include domain-specific items in their FTP measures, and (c) combine self-reports with objective outcome measures.

An issue that merits special attention in FTP research is the meaning of the term *future* in the different FTP measures. Although most of the included studies in our meta-analyses (and particularly in the education domain) defined the future in terms of length (a long-term or distant future), some studies were less clear about its meaning. For example, although Husman and Shell’s [10] FTP measure explicitly refers to the long-term future (i.e., “What one does today will have little impact on what happens ten years from now.”), Zimbardo’s measure [19] is more equivocal about the temporal distance of the future (i.e., “When I want to achieve something, I set goals and consider specific means for reaching those goals”). We believe that in order to further develop FTP theory and research it is important for FTP researchers to clarify the term future according to length and to specify its unit of time (e.g., in 5 years) within their FTP measures. This specificity regarding the time unit may also decrease the likelihood that FTP measures (as evident by its items) are confounded by individual difference variables such as locus of control and conscientiousness [56].

Finally, although our conceptual framework for coding the FTP measures offered a systematical and useful way for organizing FTP studies within the time perspective research tradition [16, 32], a recent framework from Szpunar and colleagues [213] offers another approach to code future-oriented cognitions. In contrast to our category “cognition” that includes both expectations and ideas (imagery/simulation), this framework makes a more specific distinction among future-oriented cognitions (see [213]). Future meta-analyses could incorporate these cognitions so as to deepen our understanding of the relationships between specific cognitions and behaviors.

### FTP and contextual factors

Although we have demonstrated that FTP is a significant motivator across cultures, it is important to note that these results (except for the long-term/short-term orientation dimension) are preliminary because they are only generalizable to the sample of studies included in the meta-analyses. The majority of FTP studies were conducted in Western countries (e.g., USA, Western Europe, Australia), thus, future studies should include samples from other regions as well (e.g., Asia, Eastern Europe). This would enable researchers to more deeply explore cross-cultural differences among diverse study samples.

Although Hofstede’s cultural dimensions [96] are the most widely accepted and applied dimensions when exploring culture-related variations in human behaviors in different fields of psychology (e.g., [214, 215]), and all the countries included in the meta-analyses had available sores on these dimensions, other conceptualizations of culture (e.g., Schwartz [216]; GLOBE cultural dimensions [217]) may reveal interesting cultural differences in FTP effects as well. The GLOBE future orientation dimension (“the degree to which a collectivity encourages and rewards future-oriented behaviors such as planning and delaying gratification”[217], p. 282) in particular seems relevant to be included in future FTP studies. In addition, future studies could further explore the FTP–outcome relationship as affected by other environmental factors such as socioeconomic conditions and, when student samples are involved, parental support (e.g., [218]). For example, adolescents demonstrate more optimistic perceptions regarding the future but only insofar as they receive support from their parents [219].

### FTP–outcome relationships over time

Because of a lack of longitudinal FTP studies, little is known about whether and how FTP–outcome relationships change over time. Longitudinal studies are needed, not only to test causal relationships but also to study the processes that explain the FTP–outcome relationship. Moreover, the strength of this relationship may vary with life stages or periods of transfer (e.g., stepping from adolescence to adulthood, starting a family, retiring). Although longitudinal studies are particularly needed in the work and health domains, the education domain could also benefit from longitudinal research designs; our nonsignificant results for study design in the education domain might have been due to a lack of power.

### Determinants and moderators of FTP–outcome relationships

A large majority of empirical studies have explored FTP as a precursor of attitudes and behaviors [2, 47, 48]. In contrast, studies on possible determinants of FTP have been very limited. Here we draw attention to personality characteristics that are linked to self-regulation, such as self-efficacy and feelings of control [197, 220, 221]. These variables may play a significant role in the development and content (e.g., valence) of people’s FTP.

Although we included some relevant moderators that could influence the FTP–outcome relationships, research is needed to investigate whether and in what ways other variables can moderate these relationships. For example, Fieulaine and Martinez [156] provided significant evidence for the strengthening role of desire to control (i.e., desire to maintain control, making one’s own decisions, being in charge of one’s activities) in the relationship between FTP and substance use. In addition, because FTP is essentially concerned with goal striving, it would be highly relevant to study the interaction of FTP and regulation in future goal attainment. Regulatory focus theory [222] proposes that individuals differ in the strategies they use to attain their goals—focusing on growth and advancement (promotion orientation) or focusing on safety and security (prevention orientation). A study by Joireman and colleagues [63], showed that individuals’ consideration about future consequences related positively to their healthy eating habits and exercise when they were more promotion orientated. Future research could examine how FTP and regulatory focus may interact in predicting different attitudes and behaviors among different populations (e.g., adolescents in a transition period to adulthood, adults, and retired people).

## Conclusions

We started our paper by arguing that the future is a premise of human motivation in everyday life. In the present study, we found substantial evidence that FTP is a successful motivator in three indispensable domains of life: education, work, and health. Reviewing the relationships between FTP and educational, work, and health outcomes and establishing their robustness and generalizability across life domains and cultures is a unique feature of this study. The present meta-analyses offer important results and conclusions that could be considered when developing future research on FTP. We mention here only a few. With regard to the FTP construct type, we found that FTP measures that include a combination of cognition, affect, and behavioral intention yield the strongest relationship with educational, work, and health outcomes even when controlling for identified confounds. Furthermore, we demonstrated that the FTP that involves domain-specific thinking exhibits a stronger relationship with educational outcomes than a more general FTP. Also, we showed that FTP has stronger relationships with attitudes, behavioral intention, and perceived behavioral control than with objective (verifiable) behaviors. Finally, our study identified cross-cultural differences in the strength of FTP–outcome relationships.

Our comprehensive meta-analyses add to FTP theory and research by providing the first meta-analytical evidence of the motivational force of FTP across life domains, by solving inconsistencies in study findings, and by stressing the need for further reflection on the conceptualization of FTP and its relationship with other future-oriented constructs. Ultimately, individuals’ motivation to succeed in central life domains should be built on a future that is created in their minds, hearts, and intentions.

## Supporting information

S1 File Literature search syntax. (DOCX)

S2 File Coding manual. (DOCX)

S1 Table PRISMA checklist.
(DOC)

S2 Table Subgroup analyses in education, work, and health domain. (DOCX)

S3 Table Meta-regression analyses in education, work, and health. (DOCX)

S4 Table Multiple regression models in education, work, and health. (DOCX)

S5 Table FTP and outcome type relationships in the education, work, and health domains. (DOCX)
