# Supplementary material for: Motivational power of future time perspective: Meta-analyses in education, work, and health
Source: PLoS One. 2018 Jan 24;13(1):e0190492. doi: 10.1371/journal.pone.0190492 (PMC5783357; doi:10.1371/journal.pone.0190492)
Supplement: S1 File — (DOCX) [file pone.0190492.s001.docx]

**Search Future Time Perspective**

**Databases**

PsycINFO 1.876 results (August 23, 2013)

MEDLINE 822 results (August 23, 2013)

ERIC 2.127 results (August 23, 2013)

Business Source Premier 815 results (August 23, 2013)

Web of Science 249 results (August 23, 2013)

CINAHL 445 results (August 23, 2013)

SPORTDiscus 148 results (August 23, 2013)

**Expert Authors contacted for unpublished-non-significant data from the three life domains**

4 results (February 7, 2014)

**Authors contacted for the FTP research**

(either based on the Abstract book at the 1th Time Perspective international conference (2012) or International Conference "Life designing and career counselling: Building hope and Resilience’’ (2013)

1 result (February, 21, 2014)

**Posts**

‘’Call for data for meta-analysis on Future time perspective’’ - International Time Perspective Network’’ group (LinkedIn) and the time-research listserv

12 results (February, 26, 2014)

**In total = 6.462 results; 5830 after removing the duplicates**

**After inspection of main inclusion criteria = 301**

**Collected and inserted in the Excel file and CMA3 = 65 reports/77 individual samples**

**PsycINFO**

*OvidSP*

**#1 Future time perspective**

time perspective/ OR (future adj3 time perspective*).ti,ab,id,tm. OR future time orient*.ti,ab,id,tm. OR future consequence*.ti,ab,id,tm. OR future orientation.ti,ab,id,tm. OR time orientation.ti,ab,id,tm. OR future planning.ti,ab,id,tm. OR time perspective questionnaire.ti,ab,id,tm. OR zimbardo time perspective.ti,ab,id,tm. OR (experiential time perspective ADJ1 (measure OR scale)).ti,ab,id,tm. OR wallace* measure.ti,ab,id,tm. OR Wallace* future events test.ti,ab,id,tm. OR long term personal direction scale.ti,ab,id,tm. OR achievability of future goals scale.ti,ab,id,tm. OR temporal focus scale.ti,ab,id,tm. OR (future work* adj1 (salience or goal*)).ti,ab,id,tm. OR career planning.ti,ab,id,tm. OR future career.ti,ab,id,tm.

**Results: 4.677 (August 23, 2013)**

**#2 Education domain**

learn*.ti,ab,id. OR school learning/ OR educat*.ti,ab,id. OR classroom*.ti,ab,id. OR middle school education/ OR middle school students/ OR junior high school students/ OR high school education/ OR high school students/ OR school*.ti,ab,id. OR secondary education/ OR higher education/ OR graduate education/ OR undergraduate education/ OR postgraduate training/ OR junior college students/ OR college students/ OR college*.ti,ab,id. OR academic achievement/ OR college academic achievement/ OR universit*.ti,ab,id.

**Results: 953.307 (August 23, 2013)**

**#3 Work domain**

career development/ OR career*.ti,ab,id. OR occupations/ OR occupation*.ti,ab,id. OR personnel/ OR personnel.ti,ab,id. OR job applicants/ OR job performance/ OR job*.ti,ab,id. OR work*.ti,ab,id. OR employ*.ti,ab,id. OR vocational education/ OR vocational maturity/

**Results: 693.367 (August 23, 2013)**

**#4 Health domain**

health behavior/ OR health attitudes/ OR health* behavio?r*.ti,ab,id. OR physical activity/ OR

physical activit*.ti,ab,id. OR aerobic exercise/ OR eating behavior/ OR eating attitudes/ OR eating.ti,ab,id. OR binge eating/ OR food/ OR food intake/ OR food preferences/ OR food*.ti,ab,id. OR fruit*.ti,ab,id. OR vegetable*.ti,ab,id. OR diets/ OR diet*.ti,ab,id. OR nutrition/ OR safe sex/ OR sexual risk taking/ OR safe* sex.ti,ab,id. OR sexual health.ti,ab,id. OR sexual risk.ti,ab,id. OR sexual behavio?r.ti,ab,id. OR drug abuse.ti,ab,id. OR substance abuse.ti,ab,id. OR alcohol abuse/ OR binge drinking/ OR alcohol abuse.ti,ab,id. OR smoking cessation/ OR tobacco smoking/ OR tobacco.ti,ab,id. OR smoker*.ti,ab,id. OR smoking.ti,ab,id. OR mari?uana.ti,ab,id.

**Results: 248.729 (August 23, 2013)**

**#5 Motivated behavior**

motivation/ OR motivat*.ti,ab,id. OR achievement/ OR achievement*.ti,ab,id. OR learn*.ti,ab,id. OR academic achievement motivation/ OR student engagement/ OR ((academic OR student) ADJ1 engagement).ti,ab,id. OR effort.ti,ab,id. OR student attitudes/ OR attitude*.ti,ab.OR school investment.ti,ab,id. OR attitude*.ti,ab,id. OR decision making/ OR decision*.ti,ab,id. OR choice*.ti,ab,id. OR occupational choice/ OR occupational attitudes/ OR career development/ OR vocational maturity/ OR employer attitudes/ OR aspiration*.ti,ab,id. OR health behavior/ OR health attitudes/ OR health* behavio?r*.ti,ab,id. OR physical activity/ OR physical activit*.ti,ab,id. OR aerobic exercise/ OR exercise/ OR eating behavior/ OR eating attitudes/ OR eating.ti,ab,id. OR binge eating/ OR food intake/ OR safe sex/ OR sexual risk taking/ OR safe* sex.ti,ab,id. OR sexual behavio?r.ti,ab,id. OR drug abuse.ti,ab,id. OR substance abuse.ti,ab,id. OR alcohol abuse/ OR binge drinking/ OR alcohol abuse.ti,ab,id. OR smoking cessation/ OR tobacco smoking/ OR choice behavior/ OR theory of planned behavio?r.ti,ab,id.

**Results: 984.033 (August 23, 2013)**

**1 AND (2 OR 3 OR 4) AND 5: 1.877 results (August 23, 2013)**

**MEDLINE**

*Ovid MEDLINE ® In-Process & Other Non-Indexed Citations and Ovid MEDLINE(R)*

**#1 Future time perspective**

(future adj3 time perspective*).ti,ab. OR future time orient*.ti,ab. OR future consequence*.ti,ab. OR future orientation.ti,ab. OR time orientation.ti,ab. OR future planning.ti,ab. OR time perspective questionnaire.ti,ab. OR zimbardo time perspective.ti,ab. OR (experiential time perspective ADJ1 (measure OR scale).ti,ab. OR wallace* measure.ti,ab. OR wallace* future events test.ti,ab. OR long term personal direction scale.ti,ab. OR achievability of future goals scale.ti,ab. OR temporal focus scale.ti,ab. OR (future work* adj1 (salience or goal*)).ti,ab. OR career planning.ti,ab. OR future career.ti,ab.

**Results: 1.888 (August 23, 2013)**

**#2 Education domain**

learn*.ti,ab. OR educat*.ti,ab. OR classroom*.ti,ab. OR school*.ti,ab. OR college*.ti,ab. OR universit*.ti,ab. OR students/

**Results: 963.419 (August 23, 2013)**

**#3 Work domain**

career*.ti,ab. OR occupations/ OR occupation*.ti,ab. OR personnel.ti,ab. OR job*.ti,ab. OR work*.ti,ab. OR employ*.ti,ab. OR vocational education/

**Results: 1.362.553 (August 23, 2013)**

**#4 Health domain**

health behavior/ OR attitude to health/ OR health* behavio?r*.ti,ab. OR motor activity/ OR physical activit*.ti,ab. OR eating.ti,ab. OR food/ OR food habits/ OR food preferences/ OR health food/ OR food*.ti,ab. OR fruit/ OR fruit*.ti,ab. OR vegetables/ OR vegetable*.ti,ab. OR diet/ OR diet*.ti,ab. OR safe sex/ OR safe* sex.ti,ab. OR sexual health.ti,ab. OR sexual risk.ti,ab. OR sexual behavior/ OR sexual behavio?r.ti,ab. OR drug abuse.ti,ab. OR substance abuse.ti,ab. OR alcoholism/ OR binge drinking/ OR alcohol abuse.ti,ab. OR smoking cessation/ OR smoking/ OR tobacco.ti,ab. OR smoker*.ti,ab. OR smoking.ti,ab. OR mari?uana.ti,ab.

**Results: 1.277.402 (August 23, 2013)**

**#5 Motivated behavior**

motivation/ OR motivat*.ti,ab. OR achievement/ OR achievement*.ti,ab. OR learn*.ti,ab. OR ((academic OR student) ADJ1 engagement).ti,ab. OR effort.ti,ab. OR attitude*.ti,ab. OR school investment.ti,ab. OR attitude*.ti,ab. OR decision making/ OR decision*.ti,ab. OR choice*.ti,ab. OR career choice/ OR occupational attitudes/ OR career mobility/ OR aspiration*.ti,ab. OR health behavior/ OR attitude to health/ OR health* behavio?r*.ti,ab. OR motor activity/ OR physical activit*.ti,ab. OR exercise/ OR food habits/ OR eating.ti,ab. OR safe sex/ OR safe* sex.ti,ab. OR sexual behavior/ OR sexual behavio?r.ti,ab. OR drug abuse.ti,ab. OR substance abuse.ti,ab. OR binge drinking/ OR alcohol abuse.ti,ab. OR smoking cessation/ OR choice behavior/ OR theory of planned behavio?r.ti,ab.

**Results: 1.378.153 (August 23, 2013)**

**1 AND (2 OR 3 OR 4): 1.240 results (August 22, 2013)**

**1 AND (2 OR 3 OR 4) OR 5: 822 results (August 23, 2013)**

**ERIC**

*Educational Resources Information Center (OvidSP)*

**#1 Future time perspective**

(future adj3 time perspective*).ti,ab,id. OR future time orient*.ti,ab,id. OR future consequence*.ti,ab,id. OR future orientation.ti,ab,id. OR time orientation.ti,ab,id. OR future planning.ti,ab,id. OR time perspective questionnaire.ti,ab,id. OR zimbardo time perspective.ti,ab,id. OR (experiential time perspective ADJ1 (measure OR scale).ti,ab,id. OR wallace* measure.ti,ab,id. OR Wallace* future events test.ti,ab,id. OR long term personal direction scale.ti,ab,id. OR achievability of future goals scale.ti,ab,id. OR temporal focus scale.ti,ab,id. OR (future work* adj1 (salience or goal*)).ti,ab,id. OR career planning.ti,ab,id. OR future career.ti,ab,id.

**Results: 3.298 (August 23, 2013)**

**#2 Education domain**

learn*.ti,ab. OR educat*.ti,ab. OR classroom*.ti,ab. OR middle school students/ OR junior high school students/ OR high school students/ OR school*.ti,ab. OR secondary education/ OR postsecondary education/ OR higher education/ OR undergraduate study/ OR undergraduate students/ OR graduate study/ OR graduate students/ OR college students/ OR two year college students/ OR college*.ti,ab. OR academic achievement/ OR universit*.ti,ab. OR postdoctoral education/ OR vocational education/

**Results: 1.099.219 (August 23, 2013)**

**#3 Work domain**

career development/ OR career*.ti,ab. OR occupations/ OR career choice/ OR career development/ OR occupation*.ti,ab. OR personnel.ti,ab. OR job applicants/ OR job performance/ OR job*.ti,ab. OR work*.ti,ab. OR employ*.ti,ab. OR vocational education/ OR vocational maturity/

**Results: 402.993 (August 23, 2013)**

**#4 Health domain**

health behavior/ OR health* behavio?r*.ti,ab. OR physical activities/ OR physical health/ OR physical activit*.ti,ab. OR eating habits/ OR eating.ti,ab. OR food/ OR food*.ti,ab. OR fruit*.ti,ab. OR vegetable*.ti,ab. OR diet*.ti,ab. OR nutrition/ OR sexuality/ OR safe* sex.ti,ab. OR sexual health.ti,ab. OR sexual risk.ti,ab. OR sexual behavio?r.ti,ab. OR drug abuse/ OR drug abuse.ti,ab. OR substance abuse/ OR substance abuse.ti,ab. OR alcohol abuse/ OR drinking/ OR alcohol abuse.ti,ab. OR smoking/ OR tobacco.ti,ab. OR smoker*.ti,ab. OR smoking.ti,ab. OR marijuana/ OR mari?uana.ti,ab.

**Results: 54.935 (August 23, 2013)**

**5# Motivated behavior**

motivation/ OR motivat*.ti,ab. OR achievement/ OR achievement*.ti,ab. OR learn*.ti,ab. OR academic achievement/ OR learner engagement/ OR ((academic OR student) ADJ1 engagement).ti,ab. OR effort.ti,ab. OR student motivation/ OR student attitudes/ OR attitude*.ti,ab. OR school investment.ti,ab. OR attitude*.ti,ab. OR decision making/ OR decision*.ti,ab. OR choice*.ti,ab. OR career choice/ OR career development/ OR vocational maturity/ OR employer attitudes/ OR aspiration*.ti,ab. OR health behavior/ OR health* behavio?r*.ti,ab. OR physical activities/ OR physical activit*.ti,ab. OR eating habits/ OR eating.ti,ab. OR safe* sex.ti,ab. OR sexual behavio?r.ti,ab. OR drug abuse.ti,ab. OR substance abuse.ti,ab. OR alcohol abuse/ OR drinking/ OR alcohol abuse.ti,ab. OR smoking/ OR choice behavior/ OR theory of planned behavio?r.ti,ab.

**Results: 597.612 (August 23, 2013)**

**1 AND (2 OR 3 OR 4) AND 5: 2.127 results (August 23, 2013)**

**Business Source Premier**

*EBSCO*

**#1 Future time perspective**

TI ((“future” N3 “time perspective*”) OR “future time orient*” OR “future consequence*” OR “future orientation” OR “time orientation” OR “future planning” OR “time perspective questionnaire” OR “zimbardo time perspective” OR “experiential time perspective” N1 (“measure” OR “scale”) OR “wallace* measure” OR “Wallace* future events test” OR “long term personal direction scale” OR “achievability of future goals scale” OR “temporal focus scale” OR (“future work*” N1 (“salience” OR “goal*”)) OR “career planning” OR “future career”) OR AB ((“future” N3 “time perspective*”) OR “future time orient*” OR “future consequence*” OR “future orientation” OR “time orientation” OR “future planning” OR “time perspective questionnaire” OR “zimbardo time perspective” OR “experiential time perspective” N1 (“measure” OR “scale”) OR “wallace* measure” OR “Wallace* future events test”OR “long term personal direction scale” OR “achievability of future goals scale” OR “temporal focus scale” OR (“future work*” N1 (“salience” OR “goal*”)) OR “career planning” OR “future career”) OR KW ((“future” N3 “time perspective*”) OR “future time orient*” OR “future consequence*” OR “future orientation” OR “time orientation” OR “future planning” OR “time perspective questionnaire” OR “zimbardo time perspective” OR “experiential time perspective” N1 (“measure” OR “scale”) OR “wallace* measure” OR “Wallace* future events test” OR “long term personal direction scale” OR “achievability of future goals scale” OR “temporal focus scale” OR (“future work*” N1 (“salience” OR “goal*”)) OR “career planning” OR “future career”)

**Results: 1.774 (August 23, 2013)**

**2# Motivated behavior**

DE (“employee motivation” OR “occupational achievement” OR "employees -- attitudes" OR “decision making” OR “career development” OR “employer attitudes”) OR TI (“motivat” OR “achievement*” OR “learn*” OR “effort” OR “attitude*” OR “decision*” OR “choice*” OR “aspiration*” OR “health* behavio?r*” OR “physical activit*” OR “eating” OR “safe* sex” OR “sexual behavio?r” OR “drug abuse” OR “substance abuse” OR “alcohol abuse” OR “theory of planned behavio?r”) OR AB (“motivat” OR “achievement*” OR “learn*” OR “effort” OR “attitude*” OR “decision*” OR “choice*” OR “aspiration*” OR “health* behavio?r*” OR “physical activit*” OR “eating” OR “safe* sex” OR “sexual behavio?r” OR “drug abuse” OR “substance abuse” OR “alcohol abuse” OR “theory of planned behavio?r”) OR KW (“motivat” OR “achievement*” OR “learn*” OR “effort” OR “attitude*” OR “decision*” OR “choice*” OR “aspiration*” OR “health* behavio?r*” OR “physical activit*” OR “eating” OR “safe* sex” OR “sexual behavio?r” OR “drug abuse” OR “substance abuse” OR “alcohol abuse” OR “theory of planned behavio?r”)

**Results: 1.080.777 (August 23, 2013)**

**1 AND 2: 815 (August 23, 2013)**

**Web of Science**

**#1 Future time perspective**

TS=((“future” NEAR/3 “time perspective*”) OR “future time orient*” OR “future consequence*” OR “future orientation” OR “time orientation” OR “future planning” OR “time perspective questionnaire” OR “zimbardo time perspective” OR (“experiential time perspective” NEAR/1 (“measure” OR “scale”)) OR “wallace* measure” OR “Wallace* future events test” OR “long term personal direction scale” OR “achievability of future goals scale” OR “temporal focus scale” OR (“future work*” NEAR/1 (“salience” OR “goal*”)) OR “career planning” OR “future career”)

**Results: 3.179 (August 23, 2013)**

**#2 Education domain**

TS=(“learn*” OR “school*” OR “educat*” OR “classroom*” OR “college*” OR “universit*”)

**Results: 1.304.590 (August 23, 2013)**

**#3 Work domain**

TS=(“career*” OR “occupation*” OR “personnel” OR “job*” OR “work*” OR “employ*”)

**Results: 2.465.250 (August 23, 2013)**

**#4 Health domain**

TS=(“health* behavio?r*” OR “physical activit*” OR “eating” OR “food*” OR “fruit*” OR “vegetable*” OR “diet*” OR “safe* sex” OR “sexual health” OR “sexual risk” OR “sexual behavio?r” OR “drug abuse” OR “substance abuse” OR “tobacco” OR “smoker*” OR “smoking” OR “mari?uana”)

**Results: 1.321.326 (August 23, 2013)**

**#5 Motivated behavior**

TS=(“health* behavio?r*” OR “physical activit*” OR “eating” OR “food*” OR “fruit*” OR “vegetable*” OR “diet*” OR “safe* sex” OR “sexual health” OR “sexual risk” OR “sexual behavio?r” OR “drug abuse” OR “substance abuse” OR “alcohol abuse” OR “tobacco” OR “smoker*” OR “smoking” OR “mari?uana”)

**Results: 1.328.575 (August 23, 2013)**

**1 AND (2 OR 3 OR 4) AND 5: 249 results (August 23, 2013)**

**CINAHL**

*Cumulative Index to Nursing and Allied Health Literature (EBSCO)*

**#1 Future time perspective**

TI ((“future” N3 “time perspective*”) OR “future time orient*” OR “future consequence*” OR “future orientation” OR “time orientation” OR “future planning” OR “time perspective questionnaire” OR “zimbardo time perspective” OR “experiential time perspective” N1 (“measure” OR “scale”) OR “wallace* measure” OR “Wallace* future events test” OR “long term personal direction scale” OR “achievability of future goals scale” OR “temporal focus scale” OR (“future work*” N1 (“salience” OR “goal*”)) OR “career planning” OR “future career”) OR AB ((“future” N3 “time perspective*”) OR “future time orient*” OR “future consequence*” OR “future orientation” OR “time orientation” OR “future planning” OR “time perspective questionnaire” OR “zimbardo time perspective” OR “experiential time perspective” N1 (“measure” OR “scale”) OR “wallace* measure” OR “Wallace* future events test” OR “long term personal direction scale” OR “achievability of future goals scale” OR “temporal focus scale” OR (“future work*” N1 (“salience” OR “goal*”)) OR “career planning” OR “future career”)

**Results: 837 (August 23, 2013)**

**#2 Motivated behavior**

MH (“Motivation” OR “achievement” OR “academic achievement” OR “Student Attitudes” OR “Decision Making” OR “Career Planning and Development” OR “health behavior” OR “attitude to health” OR “physical activity” OR “aerobic exercises” OR “exercise” OR “eating behavior” OR “food habits” OR “food intake” OR “safe sex” OR “alcohol abuse” OR “smoking cessation”) OR TI (“motivat” OR “achievement*” OR “learn*” OR “effort” OR “attitude*” OR “decision*” OR “choice*” OR “aspiration*” OR “health* behavio?r*” OR “physical activit*” OR “eating” OR “safe* sex” OR “sexual behavio?r” OR “drug abuse” OR “substance abuse” OR “alcohol abuse” OR “theory of planned behavio?r”) OR AB (“motivat” OR “achievement*” OR “learn*” OR “effort” OR “attitude*” OR “decision*” OR “choice*” OR “aspiration*” OR “health* behavio?r*” OR “physical activit*” OR “eating” OR “safe* sex” OR “sexual behavio?r” OR “drug abuse” OR “substance abuse” OR “alcohol abuse” OR “theory of planned behavio?r”) **Results: 391.501 (August 23, 2013)**

**1 AND 2: 445 results (August 23, 2013)**

**SPORTDiscus**

*EBSCO*

**#1 Future time perspective**

TI ((“future” N3 “time perspective*”) OR “future time orient*” OR “future consequence*” OR “future orientation” OR “time orientation” OR “future planning” OR “time perspective questionnaire” OR “zimbardo time perspective” OR “experiential time perspective” N1 (“measure” OR “scale”) OR “wallace* measure” OR “Wallace* future events test” OR “long term personal direction scale” OR “achievability of future goals scale” OR “temporal focus scale” OR (“future work*” N1 (“salience” OR “goal*”)) OR “career planning” OR “future career”) OR AB ((“future” N3 “time perspective*”) OR “future time orient*” OR “future consequence*” OR “future orientation” OR “time orientation” OR “future planning” OR “time perspective questionnaire” OR “zimbardo time perspective” OR “experiential time perspective” N1 (“measure” OR “scale”) OR “wallace* measure” OR “Wallace* future events test” OR “long term personal direction scale” OR “achievability of future goals scale” OR “temporal focus scale” OR (“future work*” N1 (“salience” OR “goal*”)) OR “career planning” OR “future career”) OR KW ((“future” N3 “time perspective*”) OR “future time orient*” OR “future consequence*” OR “future orientation” OR “time orientation” OR “future planning” OR “time perspective questionnaire” OR “zimbardo time perspective” OR “experiential time perspective” N1 (“measure” OR “scale”) OR “wallace* measure” OR “Wallace* future events test” OR “long term personal direction scale” OR “achievability of future goals scale” OR “temporal focus scale” OR (“future work*” N1 (“salience” OR “goal*”)) OR “career planning” OR “future career”)

**Results: 345 (August 23, 2013)**

**#2 Motivated behavior**

DE ("motivation (psychology)" OR "motivation in education“ OR “achievement motivation” OR “academic achievement” OR “students -- attitudes” OR “decision making” OR “career development” OR “attitude (psychology)” OR “health behavior” OR “health attitudes” OR “physical activity” OR “aerobic exercises” OR “exercise” OR “food consumption” OR “substance abuse” OR “smoking cessation”) OR TI (“motivat” OR “achievement*” OR “learn*” OR “effort” OR “attitude*” OR “decision*” OR “choice*” OR “aspiration*” OR “health* behavio?r*” OR “physical activit*” OR “eating” OR “safe* sex” OR “sexual behavio?r” OR “drug abuse” OR “substance abuse” OR “alcohol abuse” OR “theory of planned behavio?r”) OR AB (“motivat” OR “achievement*” OR “learn*” OR “effort” OR “attitude*” OR “decision*” OR “choice*” OR “aspiration*” OR “health* behavio?r*” OR “physical activit*” OR “eating” OR “safe* sex” OR “sexual behavio?r” OR “drug abuse” OR “substance abuse” OR “alcohol abuse” OR “theory of planned behavio?r”) OR KW (“motivat” OR “achievement*” OR “learn*” OR “effort” OR “attitude*” OR “decision*” OR “choice*” OR “aspiration*” OR “health* behavio?r*” OR “physical activit*” OR “eating” OR “safe* sex” OR “sexual behavio?r” OR “drug abuse” OR “substance abuse” OR “alcohol abuse” OR “theory of planned behavio?r”)

**Results: 248.247 (August 23, 2013)**

**1 AND 2: 148 results (August 23, 2013)**

**Studies from the Dissertation Abstract International**

(found by the databases syntaxes)

Two studies
