# Supplementary material for: Motivational power of future time perspective: Meta-analyses in education, work, and health
Source: PLoS One. 2018 Jan 24;13(1):e0190492. doi: 10.1371/journal.pone.0190492 (PMC5783357; doi:10.1371/journal.pone.0190492)
Supplement: S2 File — (DOCX) [file pone.0190492.s002.docx]

**Coding manual**

**Motivational power of Future time perspective: Meta-analyses in education, work, and health**

(Andre, van Vianen, Peetsma, & Oort, 2014)

Please code each study carefully in the accompanying excel sheet based on the outlined dimensions. Refer to the code and its description. Each row in the excel sheet represents an independent study. Add extra rows to the excel sheet when needed (i.e., if the study has more than one outcome type, different subsamples of a study, more than one measurement point).

| **Dimension** | **Code** |
| --- | --- |
| 1. Study ID  What is the study’s identification number? | Assign a unique identification number for each study by following the main ID of the study from the supplementary word document: “ID numbers and References of coding studies”.  In case where the paper consists of more than one independent study (i.e., consists of different samples), code each study separately. Add a letter for these different samples (e.g., 10A, 10B). In case the same sample was used, code only the study with the bigger sample size and sufficient information for coding. If a study has more than one measurement point assign to the Study ID the number of the measurement point (e.g., 11_T1, 11_T2). In case of subsamples (e.g., results for males and females separately), code each subgroup with an appropriate letter for a gender: M = males, F = females (e.g., 12_M; 12_F). |
| 2. Author/s  What is author’s surname and name? | Enter each author’s surname, and first letter of his/her name (e.g., Adams, J., & Nettle, D.). |
| 3. Study year  What is the publication year of the study? | Enter the publication year. In case the study is only online published, enter the online publication year. |
| 4. Publication status  Was the study published or unpublished? | 1 = Published study  2 = Unpublished study |
| 5. Publication type  What is the type of the publication? | 1 = Journal  2 = Book chapter  3 = Master/Doctoral dissertation  4 = Other (specify)  5 = Can’t tell |
| 6. Study design  Is the study cross-sectional or longitudinal? | 1 = Cross-sectional  2 = Longitudinal  3 = Other, specify |
| 7. Life domain  What is the life domain of the FTP scale and outcome relationship? | Code the life domain of the FTP and outcome relationship:  1 = Learning  2 = Work  3 = Health  4 = Mixed life domain (specify)  5 = Can’t tell  For example, the *FTP* and *academic achievement* relationship would be coded as number 1, as it represents the education life domain.  Please note that the relationship should belong solely to one of the three specified life domains, or their mixture (e.g., education and work). |
| 8. FTP measure name | For the purpose of this meta-analysis the authors have established a clear definition of the FTP. It is defined as *an attitude that encompasses personal cognitions*, *feelings*, and *behavioral intentions with respect to the future*. Cognitions relate to thoughts about future outcomes and goals that are valued and instrumental for current decision making and behaviors (e.g., goal planning and striving). Feelings correspond to the emotions (e.g., hope and fear) that are associated with the future, and behavioral intentions relate to individual’s plans to engage in behaviors in order to realize future goals.  In this meta-analysis we only use the FTP self-reported questionnaires as being the most dominant source of measurement and as only individuals themselves are able to report whether they reflect on the future.  Code the FTP measures which are in the line with the aforementioned definition. Type the FTP measure name, author and year (e.g., *Future scale of ZTPI* (Zimbardo & Boyd, 1999; *CFCS* (Strathman et al., 1994; *Future focus scale of Temporal focus scale* (Shipp et al., 2009). Carefully read the description of the measure and make a note if there were some changes in the current study regarding the measure (e.g., the study has used an adapted version of the scale, three items are missing, there is an extra item, etc.).  In case where the study consists of two or more FTP measures (e.g., Zimbardo’s and CFCS scale), use the one with the higher reliability reported in that study (Lipsey & Wilson, 2001).  Note that certain FTP scales and subscales are excluded from the meta-analysis as they are related to other constructs (e.g., hope, economic markers) or by having an operationalization that does not fit the FTP definition of this meta-analysis. These FTP scales and subscales are: *Delay discount rate*, Mazur, 1987*; Future Time Perspective Scale*, Carstensen & Lang, 1994, 1996, 2002; *Temporal depth scale*, Bluedorn 2002; *Speed* and *Distance (Extension)* subscale, Husman & Shell (2001); *Hope scale,* Snyder et al., 1991; *Future work self-salience* (King & Patterson, 2000, adapted by Strausss, Griffin, & Parker, 2012); *Present Time Value* and *Time Pressure* subscales from the Future-present time orientation questionnaire (Bjorgvinsson, 1999). |
| 9. Number of items in the FTP scale/subscale used  What is the number of the items in the FTP scale? | Code the number of items in the FTP scale:  1 = One to three items  2 = Four to ten  3 = Eleven and more  Please be careful when there are separate correlations per FTP subscale and the outcome of interest. In that case, please count the number of items per each FTP subscale respectively. |
| 10. FTP construct type  Which FTP component is dominant in the scale/subscale? | Similarly to Peetsma’s (2012) distinction between three FTP components (i.e., cognitive, affective, and behavioral intention), we group FTP scales based on the FTP component dominance (if more than 80 % of items belong to one component) or mixture of components (if more than 20 % are related to different components) into one of the four construct types:   1. Cognition**:** Include items about an individual’s ideas and expectations about the future. For example, “I think about what my future has in store”; “I imagine what tomorrow will bring for me.” 2. Cognition and behavioral intention: Include items about an individual’s future goals and ways to accomplish these goals (planning, setting, and self-control). For example, “When I want to get something done, I make step-by-step plans and think about how to complete each step”; “I consider how things might be in the future, and try to influence those things with my day to day behavior.” 3. Cognition and affect: Include items that focus on the affective tone of future cognitions, that is, emotions that are associated with future goals (hope, worry, fear). “If things don’t get done on time, I don’t worry about it”; “When I think about the future I feel happy.” 4. Mixture of cognition, behavioral intention, and affect: Include items that combine cognition, affect and intentions with regard to the future. For example, “I like to think of the way I will be able to develop my possibilities (capacities/talents) after school”; “I am willing to sacrifice my immediate happiness or well-being in order to achieve future outcomes.”   For example, to be coded as a FTP scale type 2, about 80 % of the items have to relate to cognitive and behavioral intention component of FTP.  Accordingly, code:  1 = Cognition  2 = Cognition and behavioral intention  3 = Cognition and affect  4 = Mixture of cognition, behavioral intention, and affect |
| 11. FTP focus  Is the FTP measure referring to a general (i.e., no specification to a certain life domain) or a specific life domain? | Code is the FTP measure referring to a general (i.e., no specification to a certain life domain) or a specific life domain:  1 = Domain general  2 = Domain specific  3 = Can’t tell |
| 12. FTP scale/subscale number  What is the number of FTP scales? | Of how many separate scales/subscales is the FTP measure consisting of:  1 = One  2 = Two (specify)  3 = Three (specify)  4 = Four or more  Enter only the number of scales/subscales that are included in the meta-analysis. |
| 13. FTP value  Is the FTP measure consisting of a separate positive and negative subscale (i.e., Future positive and Future negative)? | 1 = Yes  2 = No  3 = Can’t tell  If the answer is Yes, please pay attention to code their both effect sizes. |
| 14. Outcome name  What is the outcome/dependent variable? | Type in the precise outcome name from the study. |
| 15. Outcome measure description | Write in the name of the measure, the author’s name and the date. Write a concise description of the outcome variable with an item example. |
| 16. Outcome type  What is the outcome type based on the Theory of planned behavior (TPB; Ajzen, 1985)? | Each outcome/dependent variable of the study is to be coded based on the TPB (Ajzen, 1985). According to the TPB, human behaviors are guided by attitudes toward the behavior, normative beliefs, control beliefs, and intention (Fishbein & Ajzen, 2010). Figure 1 below implies a causal link between attitudes and behaviors that are mediated by intentions. As there were no outcomes on the normative beliefs, we exclude them from our coding. Also, as within the outcomes we noticed a distinction between behavior that is not possible to be verified and behavior that is recorded or reported, but potentially verifiable, we coded these two separately: Behavior (non-verifiable) and Behavior (verifiable).  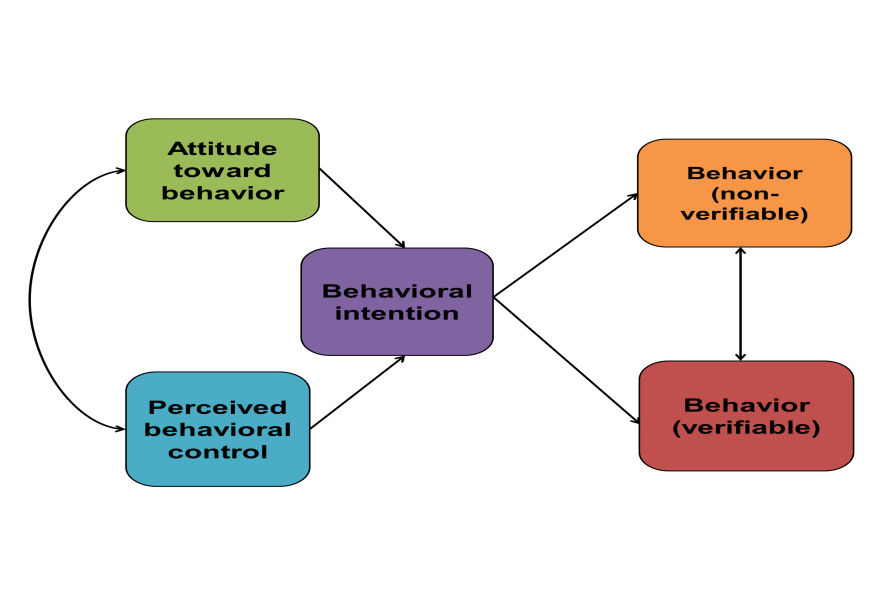  **Figure 1.** Outcome types based on the adapted TPB (Ajzen, 1985)   1. **Attitude toward behavior**   An individual’s positive or negative evaluation of self-performance of the particular behavior. The concept is the degree to which performance of the behavior is positively or negatively valued. It is determined by the total set of accessible behavioral beliefs linking the behavior to various outcomes and other attributes. In general, the more favorable the attitude towards the behavior, the stronger should be the individual’s intention to perform it. For example: ‘‘Being physically active would be . . .,’’ on a 5-point scale of useful to useless, beneficial to harmful, desirable to undesirable, good to bad, enjoyable to unenjoyable, and interesting to boring’’; Gulley, 2012).   1. **Perceived behavioral control**   An individual’s perceived ease or difficulty of performing the particular behavior (Ajzen, 1991). That is, people's perceptions of their ability to perform a given behavior. For example: ‘‘If I wanted to, I could easily be physically active on a regular basis.’’; related to controlled belief: ‘‘I believe that I have the ability to be physically active on a regular basis.’’ Gulley, 2012). Control belief or perceived behavioral control (PBC) includes individuals’ beliefs in their abilities (self-efficacy) for certain behavior and their perceptions about whether or not they will have the opportunity and resources to participate in that behavior (Fishbein & Ajzen, 2010).   1. **Behavioral intention**   An indication of an individual’s readiness to perform a given behavior. It is assumed to be an immediate antecedent of behavior (Ajzen, 2002). It is based on attitude toward the behavior, subjective norm, and perceived behavioral control, with each predictor weighted for its importance in relation to the behavior and population of interest. Intentions are assumed to capture the motivational factors that influence a behavior and to indicate how hard people are willing to try or how much effort they would exert to perform the behavior (Ajzen, 1991, p. 181). For instance: ‘’I intend to eat healthy’’; Gulley, 2012.   1. **Behavior (unverifiable):**   It is the manifest, an individual’s observable response in a given situation with respect to a given target. It is a reported behavior that cannot be verifiable. For example: ‘‘Do you participate in a school sponsored sport (s)?’’; Gulley, 2012; ''I make my supervisor aware of my work aspirations and goals''; ''Have you ever smoked cigarettes?'' ; ''Have you ever drunk alcohol (beer, wine, or liquor)''.   1. **Behavior (verifiable):**   An individual’s recorded behavior, or if reported, possible to be verifiable. For example, Grade point average, working hours as recorded or easily verifiable when self-reported, body mass index.  Accordingly code:  1 = Attitude toward behavior  2 = Perceived behavioral control  3 = Behavioral intention  4 = Behavior (unverifiable)  5 = Behavior (verifiable) |
| 17. Cultural dimension | Code each culture based on the Hofstede’s cultural dimension score available via the website:  https://www.hofstede  In case the study consists of samples from different countries, code data for each country separately (if possible). |
| 18. Sample age (mean)  What is the mean age of the sample? | Specify the mean age of the sample.  If mean age cannot be determined, enter, 999. |
| 19. Standard deviation (SD)  What is the standard deviation of the sample age (mean)? | Write down the SD of the sample age (mean).  Write 999 if the information is missing. |
| 20. Sample age group  What is the age group based on the Erikson’s (1963) stages of psychosocial development? | Code sample age group similarly to the Erikson’s stages of psychosocial development. We excluded the first three age groups (i.e., infancy; early childhood; preschool), as the FTP is being developed from about 11/12 years onwards (before this period there is fantasy). Also, we added one age group (Older adolescence). Accordingly code:   1. = School age (6 – 11 years) 2. = Adolescence (12 – 18 years) 3. = Older adolescence (19 – 23 years) 4. = Young adulthood (24 – 40 years) 5. = Middle adulthood (41 – 65 years) 6. = Maturity (66 years onwards)   Missing = 999. |
| 21. Gender  Predominant gender of the sample. | Enter the percentage of males.  Use the excel sheet made for the percentage calculation.  Enter 999 if the information is missing. |
| 22. Education level of the sample  What is the education level of the sample? | 1 = Secondary education (gymnasium, other) write down the percentage if possible  2 = University (Bachelor/master) write down the percentage if possible  If the information is missing, enter 999. |
| 23. Sample size (*N*)  What is the sample size used in the analysis? | Enter the precise sample size used in the analysis.  Enter 999 if the information is missing.  Please note that in case of longitudinal studies there is a natural attrition of the sample size at later time points. Thus, write the sample size at all measures. |
| 24. Effect size (ES) | The Pearson product-moment correlation coefficient (*r*) is to be used as the effect size index for this meta-analysis. *r* is a measure of the correlation (relationship) between the observed value of the FTP and the value of the outcome variable. For example, the strength of the relationships between FTP and job satisfaction is *r* = .25. Please note that in the case of a regression analysis, when there is one predictor variable in the model, then the standardized regression coefficient *Beta* is equivalent to the correlation coefficient (*r*) between the predictor and the criterion variable (bivariate analysis). However, when there is more than one predictor variable (multivariate regression), it is not possible to compare the contribution of each predictor variable by simply comparing the correlation coefficients. Thus, be careful and do not include these partial correlations (e.g., when in the model it is controlled for SES or other variables, more predictors in the model). Only include the correlation coefficient or the bivariate beta coefficient when it was not controlled for other variables in the model.If the study has ES for subgroups (e.g., males and females, different ethnicity; school level, different country) code them separately. Do not average. If the study has a longitudinal design use the ES from all the measurements points. In case the FTP measure consists of immediate/short-term FTP subscale (immediate) and long-term FTP (future) subscale, use the ES related to the long-term FTP subscale. When the FTP measure consists of positive and negative subscale, use the ES related from both the positive and negative FTP subscale. |
| 25. Page number ES  What is the page number where the data for the ES can be found? | Enter the page number where the ES was found. |
| 26. Effect size type  What is the ES type? | Enter the effect size type (e.g., Pearson correlation, bivariate beta coefficient, odds ratio). |
| 27. Comment | Please make a note if you encounter any difficulty or a particular issue regarding the coding. Refer precisely to the dimension’s number to which the comment is referring to. These comments will help to gain a better insight in the coding process. |
| 28. All items presence in the study  Is it possible to see all the items from the FTP measure? | Code is it possible to see all the items from the FTP measure.  In case it is not possible to see all the items, first, we will try to ask the complete measure from the author. In case this is not possible, continue coding the measure operationalisation based on the items provided in the study.  1 = I see all the items  2 = I don’t see all the items |
| 29. Sample characteristic  What is the sample characteristic? | Code the sample characteristic:  1 = General population  2 = Academically gifted  3 = Homeless people  4 = Other (specify)  5 = Can’t tell |
