# Supplementary material for: Motivational power of future time perspective: Meta-analyses in education, work, and health
Source: PLoS One. 2018 Jan 24;13(1):e0190492. doi: 10.1371/journal.pone.0190492 (PMC5783357; doi:10.1371/journal.pone.0190492)
Supplement: S2 Table — (DOCX) [file pone.0190492.s004.docx]

| Education | | | | | | | |
| --- | --- | --- | --- | --- | --- | --- | --- |
| Model | |  |  |  |  |  |  |
| Fixed |  | *Q* | *p* | *k* | *r* | LL | UL |
| FTP construct | | 27.32 | .00*** |  |  |  |  |
| Cognition | |  |  | 3 | .09 | .02 | .17 |
| Cognition and affect | |  |  | 2 | .15 | .07 | .23 |
| Cognition and beh. intention | |  |  | 11 | .23 | .20 | .26 |
| Mixture of cognition, beh. intention, and affect | |  |  | 12 | .28 | .25 | .30 |
| Random |  |  |  |  |  |  |  |
| FTP construct | | 6.93 | .07† |  |  |  |  |
| Cognition | |  |  | 3 | .10 | −.03 | .23 |
| Cognition and affect | |  |  | 2 | .16 | .00 | .31 |
| Cognition and beh. intention | |  |  | 11 | .24 | .18 | .31 |
| Mixture of cognition, beh. intention, and affect | |  |  | 12 | .28 | .22 | .34 |
| Fixed |  |  |  |  |  |  |  |
| FTP focus |  | 8.94 | .00** |  |  |  |  |
| FTP general | |  |  | 20 | .22 | .20 | .24 |
| FTP specific | |  |  | 8 | .28 | .25 | .31 |
| Random |  |  |  |  |  |  |  |
| FTP focus |  | 2.13 | .14 |  |  |  |  |
| FTP general | |  |  | 20 | .22 | .17 | .27 |
| FTP specific | |  |  | 8 | .29 | .21 | .37 |
| Fixed |  |  |  |  |  |  |  |
| Study design | | .04 | .85 |  |  |  |  |
| Cross-sectional | |  |  | 25 | .24 | .22 | .26 |
| Longitudinal | |  |  | 3 | .24 | .20 | .29 |
| Random |  |  |  |  |  |  |  |
| Study design | | .32 | .57 |  |  |  |  |
| Cross-sectional | |  |  | 25 | .24 | .20 | .29 |
| Longitudinal | |  |  | 3 | .20 | .07 | .33 |
| Work | | | | | | | |
| Model | |  |  |  |  |  |  |
| Fixed |  | *Q* | *p* | *k* | *r* | LL | UL |
|  |  |  |  |  |  |  |  |
| FTP construct | | 43.52 | .00*** |  |  |  |  |
| Cognition | |  |  | 2 | .09 | .00 | .18 |
| Cognition and beh. intention | |  |  | 6 | .19 | .14 | .24 |
| Mixture of cognition, beh. intention, and affect | |  |  | 8 | .34 | .31 | .37 |
| Random |  |  |  |  |  |  |  |
| FTP construct | | 8.39 | .02* |  |  |  |  |
| Cognition | |  |  | 2 | .11 | −.10 | .31 |
| Cognition and beh. intention | |  |  | 6 | .15 | .04 | .27 |
| Mixture of cognition, beh. intention, and affect | |  |  | 8 | .34 | .25 | .43 |
| Health | | | | | | | |
| Model | |  |  |  |  |  |  |
| Fixed |  | *Q* | *p* | *k* | *r* | LL | UL |
| FTP construct | | 8.83 | .00** |  |  |  |  |
| Cognition and beh. intention | |  |  | 20 | .18 | .16 | .20 |
| Mixture of cognition, beh. intention, and affect | |  |  | 11 | .23 | .20 | .25 |
| Random |  |  |  |  |  |  |  |
| FTP construct | | 1.61 | .20 |  |  |  |  |
| Cognition and beh. intention | |  |  | 20 | .19 | .15 | .22 |
| Mixture of cognition, beh. intention, and affect | |  |  | 11 | .23 | .18 | .27 |
| Fixed |  |  |  |  |  |  |  |
| FTP focus |  | .25 | .62 |  |  |  |  |
| FTP general | |  |  | 28 | .20 | .18 | .22 |
| FTP specific | |  |  | 4 | .22 | .14 | .30 |
| Random |  |  |  |  |  |  |  |
| FTP focus |  | .04 | .84 |  |  |  |  |
| FTP general | |  |  | 28 | .21 | .17 | .24 |
| FTP specific | |  |  | 4 | .22 | .11 | .32 |
| *Note.* *k* = number of studies; *r* = effect size; LL = lower limit; UL = upper limit.  †*p* < .10. **p* < .05. ***p* < .01. ****p* < .0001. | | | | |  |  |  |
