# Supplementary material for: Motivational power of future time perspective: Meta-analyses in education, work, and health
Source: PLoS One. 2018 Jan 24;13(1):e0190492. doi: 10.1371/journal.pone.0190492 (PMC5783357; doi:10.1371/journal.pone.0190492)
Supplement: S3 Table — (DOCX) [file pone.0190492.s005.docx]

| Life domain | Model | Moderator |  | |  |  | B | *SE* | *p* |
| --- | --- | --- | --- | --- | --- | --- | --- | --- | --- |
| Education | Fixed | Culture–individualism/collectivism | | | | | .0007 | .0005 | .18 |
|  |  | Culture–long-term/short-term | | | |  | .0021 | .0005 | .0001*** |
|  |  | Culture–uncertainty-avoidance | | | |  | .001 | .0006 | .09† |
|  |  | Culture–indulgence/restraint | | | |  | .0001 | .0006 | .91 |
|  |  | Age |  | |  |  | .00 | .0035 | .38 |
|  |  | Gender |  | |  |  | .0013 | .0007 | .05* |
|  |  | Publication year | | |  |  | .0002 | .0027 | .93 |
|  | Random | Culture–individualism/collectivism | | | | | .0006 | .0011 | .60 |
|  |  | Culture–long-term/short-term | | | |  | .0022 | .0011 | .03* |
|  |  | Culture–uncertainty-avoidance | | | |  | .001 | .0013 | .42 |
|  |  | Culture–indulgence/restraint | | | |  | −.0002 | .0014 | .90 |
|  |  | Age |  | |  |  | −.0051 | .0078 | .51 |
|  |  | Gender |  | |  |  | .0009 | .0014 | .53 |
|  |  | Publication year | | |  |  | .0017 | .0063 | .78 |
| Work | Fixed | Culture–individualism/collectivism | | | |  | .001 | .0006 | .07† |
|  |  | Culture–long-term/short-term | | | |  | −.0003 | .0005 | .56 |
|  |  | Culture–uncertainty-avoidance | | | |  | .0001 | .0006 | .85 |
|  |  | Culture–indulgence/restraint | | | |  | −.0036 | .0008 | .0001*** |
|  |  | Age |  | |  |  | −.0052 | .0013 | .0001*** |
|  |  | Gender |  | |  |  | .0036 | .001 | .0004** |
|  |  | Publication year | | |  |  | .0036 | .0023 | .10 |
|  | Random | Culture–individualism/collectivism | | | | | .0002 | .002 | .93 |
|  |  | Culture–long-term/short-term | | | |  | .0013 | .002 | .50 |
|  |  | Culture–uncertainty-avoidance | | | |  | .001 | .0018 | .56 |
|  |  | Culture–indulgence/restraint | | | |  | −.0029 | .0022 | .18 |
|  |  | Age |  | |  |  | −.0013 | .0037 | .72 |
|  |  | Gender |  | |  |  | .0018 | .0035 | .61 |
|  |  | Publication year | | |  |  | .0054 | .0048 | .26 |
| Health | Fixed | Culture–individualism/collectivism | | | | | .0031 | .0011 | .004* |
|  |  | Culture–long-term/short-term | | | |  | .0007 | .0006 | .29 |
|  |  | Culture–uncertainty-avoidance | | | |  | .0005 | .0007 | .52 |
|  |  | Culture–indulgence/restraint | | | |  | .0006 | .0007 | .44 |
|  |  | Age |  | |  |  | −.0016 | .0009 | .09† |
|  |  | Gender |  | |  |  | −.0026 | .0007 | .0001*** |
|  |  | Publication year | | |  |  | .0044 | .0015 | .003** |
|  | Random | Culture–individualism/collectivism | | | | | .0033 | .002 | .10 |
|  |  | Culture–long term/short term | | | |  | .0011 | .0012 | .36 |
|  |  | Culture–uncertainty-avoidance | | | |  | .0006 | .0014 | .65 |
|  |  | Culture–indulgence/restraint | | | |  | .0012 | .0018 | .51 |
|  |  | Age |  | |  |  | −.0015 | .0018 | .42 |
|  |  | Gender |  | |  |  | −.0014 | .0011 | .18 |
|  |  | Publication year | | |  |  | .0028 | .0029 | .34 |
| *Note.* B = unstandardized beta weight; *SE* = standard error. | | | | | |  |  |  |  |
| †*p* < .10. **p* < .05. ***p* < .001. ****p* < .0001. | | | |  |  |  |  |  |  |
