# Supplementary material for: Motivational power of future time perspective: Meta-analyses in education, work, and health
Source: PLoS One. 2018 Jan 24;13(1):e0190492. doi: 10.1371/journal.pone.0190492 (PMC5783357; doi:10.1371/journal.pone.0190492)
Supplement: S4 Table — (DOCX) [file pone.0190492.s006.docx]

|  | | | *Q* | *df* | *p* | B | *SE* | *p* |
| --- | --- | --- | --- | --- | --- | --- | --- | --- |
| Domain | Model | Moderators |  |  |  |  |  |  |
| Education | Fixed | FTP construct type | 20 | 3 | .0002** |  |  |  |
|  |  | FTP focus |  |  |  | −.06 | .03 | .07† |
|  |  | Culture–long-term orientation |  |  |  | .00 | .00 | .61 |
|  |  | Culture–uncertainty-avoidance |  |  |  | .00 | .00 | .11 |
|  |  | Gender |  |  |  | .00 | .00 | .21 |
|  | Random | FTP construct type | 5.66 | 3 | 0.13 |  |  |  |
|  |  | FTP focus |  |  |  | −.08 | .08 | .32 |
|  |  | Culture–long-term orientation |  |  |  | .00 | .00 | .81 |
|  |  | Culture–uncertainty-avoidance |  |  |  | .00 | .00 | .57 |
|  |  | Gender |  |  |  | .00 | .00 | .63 |
|  | *R*^2^ | .08 |  |  |  |  |  |  |
| Work | Fixed | FTP construct type | 40.27 | 3 | .0001** |  |  |  |
|  |  | Culture–individualism/collectivism | |  |  | −.01 | .00 | .001** |
|  |  | Culture–indulgence/restraint |  |  |  | .00 | .00 | .16 |
|  |  | Age |  |  |  | .00 | .00 | .11 |
|  | Random | FTP construct type | 11.21 | 3 | .01* |  |  |  |
|  |  | Culture–individualism/collectivism | |  |  | −.01 | .00 | .1 |
|  |  | Culture–indulgence/restraint |  |  |  | .00 | .00 | .62 |
|  |  | Age |  |  |  | .01 | .01 | .2 |
|  | *R*^2^ | .11* |  |  |  |  |  |  |
| Health | Fixed | FTP construct type | 13.75 | 2 | .001* |  |  |  |
|  |  | Culture–individualism/collectivism | |  |  | .00 | .00 | .04* |
|  |  | Gender |  |  |  | .00 | .00 | .02* |
|  |  | Year of publication |  |  |  | .01 | .00 | .01* |
|  | Random | FTP construct type | 8.03 | 2 | .02* |  |  |  |
|  |  | Culture–individualism/collectivism | |  |  | .00 | .00 | .38 |
|  |  | Gender |  |  |  | .00 | .00 | .32 |
|  |  | Year of publication |  |  |  | .00 | .00 | .26 |
|  | *R*^2^ | .30* |  |  |  |  |  |  |
| *Note.* B = unstandardized beta weight; *SE* = standard error. | | | |  |  |  |  |  |
| † *p* < .10. **p* < .05. ***p* < .001. | | |  |  |  |  |  |  |
