# Supplementary material for: Motivational power of future time perspective: Meta-analyses in education, work, and health
Source: PLoS One. 2018 Jan 24;13(1):e0190492. doi: 10.1371/journal.pone.0190492 (PMC5783357; doi:10.1371/journal.pone.0190492)
Supplement: S5 Table — (DOCX) [file pone.0190492.s007.docx]

| Education | | | | | | | | | | | | | | | | | | | | | | |
| --- | --- | --- | --- | --- | --- | --- | --- | --- | --- | --- | --- | --- | --- | --- | --- | --- | --- | --- | --- | --- | --- | --- |
|  |  |  | Effect size and 95% interval | | |  | Test of null (2-Tail) | |  | Heterogeneity | | | | | | | |  | Tau-squared | | | |
|  |  | *k* | *r* | LL | UL |  | Z | *p* |  | *Q* | | *df* (*Q*) | | | *p* | | *I^2^* |  | T^2^ | *SE* | *σ* | T |
| FTP and ATB |  |  |  |  |  |  |  |  |  |  | |  | | |  | |  |  |  |  |  |  |
| Fixed |  | 7 | .28 | .24 | .32 |  | 14.65 | .00**** |  | 25.51 | | 6.00 | | | .00 | | 76.48 |  | .01 | .01 | .00 | .10 |
| Random |  | 7 | .29 | .21 | .36 |  | 7.00 | .00**** |  |  | |  | | |  | |  |  |  |  |  |  |
| FTP and BI |  |  |  |  |  |  |  |  |  |  | |  | | |  | |  |  |  |  |  |  |
| Fixed |  | 4 | .23 | .18 | .27 |  | 9.99 | .00**** |  | 68.97 | | 3.00 | | | .00 | | 95.65 |  | .05 | .05 | .00 | .23 |
| Random |  | 4 | .28 | .06 | .48 |  | 2.52 | .01** |  |  | |  | | |  | |  |  |  |  |  |  |
| FTP and PBC |  |  |  |  |  |  |  |  |  |  | |  | | |  | |  |  |  |  |  |  |
| Fixed |  | 3 | .26 | .21 | .32 |  | 9.17 | .00**** |  | 9.80 | | 2.00 | | | .01 | | 79.59 |  | .01 | .02 | .00 | .11 |
| Random |  | 3 | .25 | .12 | .37 |  | 3.60 | .00*** |  |  | |  | | |  | |  |  |  |  |  |  |
| FTP and UB |  |  |  |  |  |  |  |  |  |  | |  | | |  | |  |  |  |  |  |  |
| Fixed |  | 12 | .34 | .31 | .37 |  | 21.99 | .00**** |  | 30.68 | | 11.00 | | | .00 | | 64.14 |  | .01 | .00 | .00 | .08 |
| Random |  | 12 | .33 | .28 | .38 |  | 11.39 | .00**** |  |  | |  | | |  | |  |  |  |  |  |  |
| FTP and VB |  |  |  |  |  |  |  |  |  |  | |  | | |  | |  |  |  |  |  |  |
| Fixed |  | 14 | .16 | .14 | .19 |  | 12.01 | .00**** |  | 48.51 | | 13.00 | | | .00 | | 73.20 |  | .01 | .00 | .00 | .09 |
| Random |  | 14 | .16 | .11 | .21 |  | 5.81 | .00**** |  |  | |  | | |  | |  |  |  |  |  |  |
| Work | | | | | | | | | | | | | | | | | | | | | | |
|  |  |  | Effect size and 95% interval | | |  | Test of null (2-Tail) | |  | | Heterogeneity | | | | | | |  | Tau-squared | | | |
|  |  | *k* | *r* | LL | UL |  | Z | *p* |  | | *Q* | | | *df* (*Q*) | | *p* | *I^2^* |  | T^2^ | *SE* | *σ* | T |
| FTP and ATB |  |  |  |  |  |  |  |  |  | |  | | |  | |  |  |  |  |  |  |  |
| Fixed |  | 12 | .22 | .20 | .25 |  | 18.08 | .00**** |  | | 36.96 | | | 11.0 | | .00 | 70.24 |  | .01 | .00 | .00 | .08 |
| Random |  | 12 | .21 | .16 | .27 |  | 7.74 | .00**** |  | |  | | |  | |  |  |  |  |  |  |  |
| FTP and BI |  |  |  |  |  |  |  |  |  | |  | | |  | |  |  |  |  |  |  |  |
| Fixed |  | 7 | .00 | -.03 | .03 |  | .09 | .93 |  | | 474.38 | | | 6.00 | | .00 | 98.74 |  | .15 | .12 | .01 | .38 |
| Random |  | 7 | .20 | −.08 | .46 |  | 1.42 | .16 |  | |  | | |  | |  |  |  |  |  |  |  |
| FTP and PBC |  |  |  |  |  |  |  |  |  | |  | | |  | |  |  |  |  |  |  |  |
| Fixed |  | 9 | .33 | .31 | .35 |  | 2.52 | .00**** |  | | 42.05 | | | 8.00 | | .00 | 80.98 |  | .01 | .01 | .00 | .10 |
| Random |  | 9 | .32 | .25 | .38 |  | 8.68 | .00**** |  | |  | | |  | |  |  |  |  |  |  |  |
| FTP and UB |  |  |  |  |  |  |  |  |  | |  | | |  | |  |  |  |  |  |  |  |
| Fixed |  | 5 | .39 | .35 | .43 |  | 17.15 | .00**** |  | | 106.75 | | | 4.00 | | .00 | 96.25 |  | .08 | .06 | .00 | .28 |
| Random |  | 5 | .45 | .23 | .62 |  | 3.76 | .00**** |  | |  | | |  | |  |  |  |  |  |  |  |
| FTP and VB |  |  |  |  |  |  |  |  |  | |  | | |  | |  |  |  |  |  |  |  |
| Fixed |  | 2 | .00 | −.09 | .08 |  | −.09 | .93 |  | | 12.79 | | | 1.00 | | .00 | 92.18 |  | .06 | .08 | .01 | .23 |
| Random |  | 2 | −.08 | −.39 | .26 |  | −.44 | .66 |  | |  | | |  | |  |  |  |  |  |  |  |
| Health | | | | | | | | | | | | | | | | | | | | | | |
|  |  |  | Effect size and 95% interval | | |  | Test of null (2-Tail) | |  | | Heterogeneity | | | | | | |  | Tau-squared | | | |
|  |  | *k* | *r* | LL | UL |  | Z | *p* |  | | *Q* | | *df* (*Q*) | | *p* | | *I^2^* |  | T^2^ | *SE* | *σ* | T |
| FTP and ATB |  |  |  |  |  |  |  |  |  | |  | |  | |  | |  |  |  |  |  |  |
| Fixed |  | 8 | .13 | .08 | .17 |  | 5.39 | .00**** |  | | 13.16 | | 7.00 | | .07 | | 46.79 |  | .00 | .00 | .00 | .06 |
| Random |  | 8 | .14 | .08 | .21 |  | 4.22 | .00**** |  | |  | |  | |  | |  |  |  |  |  |  |
| FTP and BI |  |  |  |  |  |  |  |  |  | |  | |  | |  | |  |  |  |  |  |  |
| Fixed |  | 9 | .24 | .21 | .27 |  | 14.75 | .00**** |  | | 24.69 | | 8.00 | | .00 | | 67.59 |  | .01 | .01 | .00 | .08 |
| Random |  | 9 | .21 | .15 | .28 |  | 6.34 | .00**** |  | |  | |  | |  | |  |  |  |  |  |  |
| FTP and PBC |  |  |  |  |  |  |  |  |  | |  | |  | |  | |  |  |  |  |  |  |
| Fixed |  | 6 | .17 | .13 | .22 |  | 7.16 | .00**** |  | | 8.66 | | 5.00 | | .12 | | 42.26 |  | .00 | .00 | .00 | .05 |
| Random |  | 6 | .17 | .11 | .24 |  | 5.12 | .00**** |  | |  | |  | |  | |  |  |  |  |  |  |
| FTP and UB |  |  |  |  |  |  |  |  |  | |  | |  | |  | |  |  |  |  |  |  |
| Fixed |  | 26 | .19 | .17 | .20 |  | 20.35 | .00**** |  | | 69.88 | | 25.0 | | .00 | | 64.22 |  | .00 | .00 | .00 | .07 |
| Random |  | 26 | .21 | .18 | .24 |  | 12.06 | .00**** |  | |  | |  | |  | |  |  |  |  |  |  |
| FTP and VB |  |  |  |  |  |  |  |  |  | |  | |  | |  | |  |  |  |  |  |  |
| Fixed |  | 3 | .14 | .08 | .19 |  | 4.59 | .00**** |  | | 1.20 | | 2.00 | | .55 | | .00 |  | .00 | .00 | .00 | .00 |
| Random |  | 3 | .14 | .08 | .19 |  | 4.59 | .00**** |  | |  | |  | |  | |  |  |  |  |  |  |
| *Note.* ATB = attitude toward behavior; BI = behavioral intention; PBC = perceived behavioral control; UB = unverifiable behavior; VB = verifiable behavior; *k =* number of studies; *r* = effect size; LL = lower limit; UL = upper limit.  **p* < .05. ***p* < .01. ****p* < .001. *****p* < .0001. | | | | | | | | | | | | | | | | | | | | | | |
